# Intelligent Omni-Surface-Assisted Cooperative Hybrid Non-Orthogonal Multiple Access: Enhancing Spectral Efficiency Under Imperfect Successive Interference Cancellation and Hardware Distortions

**DOI:** 10.3390/s25072283

**Published:** 2025-04-03

**Authors:** Helen Sheeba John Kennedy, Vinoth Babu Kumaravelu

**Affiliations:** Department of Communication Engineering, School of Electronics Engineering, Vellore Institute of Technology, Vellore 632014, Tamil Nadu, India; helensheeba.j2022@vitstudent.ac.in

**Keywords:** cooperative communication, intelligent omni-surfaces (IOSs), imperfect successive interference cancellation (iSIC), hardware distortions (HWDs), outage probability, sum spectral efficiency (SSE)

## Abstract

Non-orthogonal multiple access (NOMA) has emerged as a key enabler of massive connectivity in next-generation wireless networks. However, conventional NOMA studies predominantly focus on two-user scenarios, limiting their scalability in practical multi-user environments. A critical challenge in these systems is error propagation in successive interference cancellation (SIC), which is further exacerbated by hardware distortions (HWDs). Hybrid NOMA (HNOMA) mitigates SIC errors and reduces system complexity, yet cell-edge users (CEUs) continue to experience degraded sum spectral efficiency (SSE) and throughput. Cooperative NOMA (C-NOMA) enhances CEU performance through retransmissions but incurs higher energy consumption. To address these limitations, this study integrates intelligent omni-surfaces (IOSs) into a cooperative hybrid NOMA (C-HNOMA) framework to enhance retransmission efficiency and extend network coverage. The closed-form expressions for average outage probability and throughput are derived, and a power allocation (PA) optimization framework is proposed to maximize SSE, with validation through Monte Carlo simulations. The introduction of a novel strong–weak strong–weak (SW-SW) user pairing strategy capitalizes on channel diversity, achieving an SSE improvement of ∼0.48% to ∼3.81% over conventional pairing schemes. Moreover, the proposed system demonstrates significant performance gains as the number of IOS elements increases, even under imperfect SIC (iSIC) and HWD conditions. By optimizing PA values, SSE is further enhanced by at least 2.24%, even with an SIC error of 0.01 and an HWD level of 8%. These results underscore the potential of an IOS-assisted C-HNOMA system with SW-SW pairing as a viable solution for improving multi-user connectivity, SSE, and system robustness in future wireless communication networks.

## 1. Introduction

The need for exponential improvements in data rate, reliability, throughput, and massive connectivity drives the transition from fifth-generation (5G) to sixth-generation (6G) systems. While 5G offers peak data rates of up to 20 Gbps and significantly reduced latency, 6G aims to achieve data rates exceeding 100 Gbps, with even lower latency, near-instantaneous communication, and enhanced reliability [1]. Additionally, 6G also promises to support a vast number of connected devices, ensuring massive connectivity in diverse environments. This leap will support unprecedented connectivity and data-hungry applications, including augmented reality, remote surgery, and industrial automation, fostering an era of seamless and ultra-reliable communication networks [1,2].

The conventional orthogonal multiple access (OMA) techniques assign distinct resources to individual user equipment (UE), limiting the number of simultaneous UE. This limitation poses a significant challenge for accommodating a large number of UE, as the available resources are quickly depleted with increasing device counts. In contrast, the non-orthogonal multiple access (NOMA) method enables different UE typesto share the same resources through power domain and code domain techniques, thereby substantially enhancing the supported UE [3]. However, this advantage comes with the trade-off of heightened complexity in signal processing and potential successive interference cancellation (SIC) issues [4].

NOMA’s complexity arises primarily from the need for SIC to differentiate between UE signals, which is computationally intensive and challenging to implement effectively. Additionally, in practical scenarios, the UE often faces hardware distortions (HWDs) that can further degrade performance [5]. In conventional NOMA, as the UE increases, the complexity arising from SIC also increases. Therefore, with conventional NOMA, the UE experiences large outages, less sum spectral efficiency (SSE), and may not meet area traffic capacity (bps/km^2^). In a practical scenario, the perfect SIC (pSIC) is not possible. Therefore, errors occur due to imperfect SIC (iSIC), and for large UE, this error is propagated and the complexity increases, resulting in performance degradation. These drawbacks can be mitigated by employing hybrid NOMA (HNOMA) systems, which combine the advantages of both OMA and NOMA. HNOMA schemes can leverage the benefits of resource allocation from OMA while still utilizing the enhanced connectivity and capability of NOMA, providing a more robust and efficient solution for massive connectivity in real-world environments [6]. HNOMA acts as a simple two-UE NOMA system within every sub-frame. Therefore, the degradation of performance due to iSIC and HWDs is compensated for and, indirectly, HNOMA reduces the SIC error propagation.

Through the NOMA phenomenon, larger power is allocated to weaker UE (WUE), and the remaining small power fraction is given to stronger UE (SUE) to maintain fairness. However, to support the quality of service (QoS) of each UE, the power allocation (PA) has to be optimized. Despite giving large power to the WUE, there exists a degradation in WUE performance. This is due to the weaker link between next-generation NodeB (gNB) and cell-edge UE.

Cooperative NOMA (C-NOMA) utilizes the natural synergy within NOMA. In this approach, the SUE decodes the WUE’s data and relays them, providing signal diversity [7]. This dual-link transmission significantly enhances the performance of the WUE in the system. This results in diversity gain without requiring additional antennas, as seen in multi-input multi-output (MIMO) systems. Furthermore, cooperative relaying can effectively extend the coverage area of the gNB, ensuring more reliable and extensive communication with enhanced outage probability and throughput performance, especially for UE with poor channel conditions [8,9]. However, the SUE has to perform a retransmission to the WUE. This requires large power from the SUE, which eventually drains the SUE’s battery. During the integration of reconfigurable intelligent surfaces (RISs) during retransmission/cooperative relaying, the SUE does not require much power, and it can also reach cell-edge UE and UE at dead zones with the least power [10,11].

RISs are capable of dynamically manipulating electromagnetic waves to enhance signal quality and coverage [12,13]. However, their limitation lies in their half-space coverage, which restricts their effectiveness in environments requiring comprehensive signal reach. In contrast, intelligent omni-surfaces (IOSs) overcome this drawback by offering full space coverage, ensuring seamless connectivity in all directions [13,14]. IOSs offer comprehensive coverage by reflecting and refracting signals omnidirectionally, unlike RISs, which direct signals in one way. This enhances network flexibility, especially in complex environments, enabling seamless communication for multiple users both indoors and outdoors. While RISs may cause dead zones, IOSs ensure effective signal transmission and adapt to varied situations using multipath propagation, boosting coverage and reliability. It maintains strong link stability by adjusting to environmental changes, critical in highly mobile or obstructed areas. Although RISs perform well in controlled environments, it struggles in dynamic, crowded spaces requiring real-time adjustments. This expansive coverage makes the IOS more suitable for complex and dynamic communication scenarios, providing a more versatile and reliable solution for future wireless networks [15,16].

IOSs offer several advantages over RISs, making it a promising technology for future wireless communication systems. However, IOS also presents certain challenges, including self-interference caused by multipath effects, coupling losses, and non-linear distortions [17]. These interferences can lead to power leakage, correlated interference, and constructive or destructive interference, ultimately degrading overall system performance. To mitigate these challenges, IOS component design can be optimized with high-isolation architectures to minimize mutual coupling effects. Advanced signal processing techniques at the radio frequency (RF) chains, such as RF cancellation using analog circuits, can effectively reduce correlated interference [18]. Additionally, integrating hybrid active–passive IOS components with adaptive filters within the architecture can help compensate for losses due to non-linear distortions, enhancing overall system efficiency and reliability [19].

The proposed IOS-aided C-HNOMA aims to enhance coverage and ensure reliable connectivity in congested networks, making it ideal for smart cities and surveillance systems. It also supports smart agriculture by maintaining internet of things (IoT) sensor connectivity in remote fields [2]. The improved coverage offered by this system facilitates seamless communication, while HNOMA reduces the complexity associated with massive connectivity, thereby boosting network efficiency. In urban environments, IOS-aided C-HNOMA can be utilized in smart streetlights and surveillance systems to enable uninterrupted data transmission. Additionally, HNOMA allows unmanned aerial vehicles (UAVs) to function as relays, providing coverage in disaster areas or remote locations lacking infrastructure [17,20]. Thus, the proposed IOS-aided C-HNOMA has the potential to revolutionize IoT applications, smart cities, vehicle-to-everything (V2X) communication, and UAV functionality by improving spectral efficiency (SE), coverage, and energy efficiency [21]. It enables intelligent signal control for reliable connectivity in dynamic environments, positioning itself as a crucial enabler for future wireless networks.

### Organization

This paper is structured as follows: Section 2 presents a comprehensive literature review, highlighting recent advancements in NOMA and C-NOMA, iSIC and HWD effects, HNOMA, and RIS and IOS technologies. Section 3 outlines the system model, describing the pairing schemes, IOS integration, and considerations for iSIC and HWDs. Section 4 delves into the performance analysis, deriving analytical expressions for the outage probability, throughput, diversity order, and SSE and quantifying the IOS’s impact. Section 5 discusses the simulation results, validates the derived analytical expressions, and shows the enhancement in the performance of the proposed system. Finally, Section 6 concludes the manuscript by summarizing key findings and suggesting directions for future research.

## 2. Related Works

This section discusses the significant advancements in wireless communication technologies, particularly in NOMA and its variants. It focuses on the following areas: HNOMA and C-NOMA systems, the effect of iSIC and HWDs, RIS-aided NOMA, and IOS-aided systems. This analysis highlights the research gaps and the contributions of the proposed work.

### 2.1. NOMA for Dense Network Connectivity

NOMA is a leading candidate technology for extensive connectivity to next-generation networks. The authors of [3] explore power-domain NOMA, focusing on optimizing PA to achieve proportional fairness and minimize outage probabilities under block fading conditions. The authors evaluated system throughput and latency using hybrid automatic repeat request (HARQ) protocols and maximum ratio combining (MRC), providing numerical results for both symmetric and asymmetric scenarios. In [22], the PA optimization in NOMA systems to enhance SSE and UE fairness is investigated. The study compares three metaheuristic algorithms—differential evolution (DE), particle swarm optimization (PSO), and artificial bee colony (ABC)—to solve the non-convex optimization problem. The results show that DE optimization maximizes SSE by ∼1.8% to ∼10% with lower complexity, reducing signal-to-noise ratio (SNR) requirements for UE.

The authors of [6] explored the performance of integrated sensing and communication (ISAC) systems using NOMA and signal alignment techniques. The study investigates different precoding designs, such as sensing-centric, communications-centric, and Pareto optimal, and evaluates key performance metrics such as communication rate and outage probability. Numerical results demonstrate that ISAC outperforms conventional frequency-division sensing in terms of spectrum, energy, and hardware efficiency, showcasing its superiority in achieving a broader rate region. The authors of [23] suggested a joint design framework that leverages the generalized polarization effect among UE, decomposing the NOMA channel into multiple-bit polarized channels. The recommended schemes, sequential UE partition and parallel UE partition, improve system performance by optimizing the NOMA decoding order and reducing latency.

### 2.2. C-NOMA for Reliable Network Connectivity

This section further elaborates on the advantages of integrating cooperative communication into the NOMA system. The authors of [7] addressed the challenge of ensuring secrecy fairness in downlink (DL) C-NOMA systems with untrusted receivers and iSIC. The study suggested joint PA and decoding order optimization to maximize the minimum secrecy rate among UE. The authors of [8] focused on an SUE that assists the WUE by relaying information while harvesting energy using a battery-assisted model. The authors derived closed-form expressions for outage probabilities and evaluated the impact of self-interference and energy harvesting (EH) protocols.

The authors of [9] explored a game-theoretic framework to address the challenges of PA and subcarrier assignment, aiming to elevate the overall system performance. The authors presented algorithms enabling UE distributed decision-making, leading to improved SE and fairness. The authors of [24] investigated the bit error rate (BER) and ergodic SSE (E-SSE) of the suggested spatial modulation (SM)-C-NOMA system. The results demonstrate that the SM-C-NOMA system achieves superior BER and E-SSE performance.

### 2.3. HNOMA for Reduced HWD and SIC Complexity

This section discusses HNOMA systems’ significance in reducing complexity and propagation errors. In [4], the authors analyzed the outage probability of NOMA systems under iSIC conditions. The closed-form expressions are derived for the outage probability of the Rician fading channel model, approximating with a Gamma distribution to simplify calculations. A low-complexity PA strategy is introduced to maximize SSE, achieving improvements of about ∼4.14% to ∼6.19% in DL and uplink scenarios. The authors of [5] introduced opportunistic UE to share time slots with legacy UE, improving transmission opportunities. The study presented a power-reducing coefficient to ensure lower energy consumption than conventional OMA under HWD and iSIC conditions. Section 3.1 elaborates on the pairing strategies used in the HNOMA system.

### 2.4. RIS-Assisted NOMA

This section details the RIS for wide coverage in the NOMA system. The authors of [10] investigated the performance of a pair of NOMA UE over Nakagami-*m* fading channels. It derives expressions for outage probabilities with both pSIC and iSIC schemes. The authors of [15] explored partial RIS selection (PRIS) to analyze outage probabilities and throughput for two UE systems. The findings show that PRIS can enhance user fairness and improve system performance compared to conventional NOMA systems without RISs.

The authors of [11] suggested a novel ISAC system using NOMA and a dedicated RIS providing virtual line-of-sight (LoS) links for radar targets, addressing path loss and blockage. The study optimized the beamforming technique to maximize the beampattern gain. For a given PA, active beamforming, and RIS phase values, the authors of [25] developed a max-min problem to optimize the sensing beam pattern with SE constraints. The recommended low-complexity alternate optimization (AO) algorithm demonstrates improved beampattern gain. The authors of [26] investigated the performance of active RISs in NOMA networks considering HWDs.

In [12], an RIS-assisted NOMA system based on space shift keying (SSK) is introduced to leverage RISs to enhance DL transmission by optimizing phase shifts, resulting in significant gains in BER performance. The suggested system outperforms conventional RIS-assisted OMA systems by ∼10 to ∼20 dB under similar conditions. The authors of [13] explored UE pairing strategies for DL RIS-assisted HNOMA. The study delves into optimizing UE pairing to enhance communication efficiency and reliability. The findings suggested that RIS-assisted HNOMA significantly enhances SSE in high-SNR regions.

### 2.5. IOS-Assisted NOMA

This section discusses the advancement of IOSs in the NOMA system. The authors of [14] examined the performance of IOS-assisted DL NOMA networks with phase quantization and channel estimation errors. Simulation results show that IOS-assisted NOMA performs comparably to OMA with negligible impact from channel correlation for larger numbers of IOS components (L). The authors of [16] investigated using IOSs under millimeter wave (mmWave) networks. The study formulated the SSE maximization problem and suggested an AO framework to design active and passive beamforming vectors and PA factors.

In [20], the performance of vehicle-to-vehicle (V2V) communications using RISs and IOSs under NOMA and OMA schemes is investigated. The study derived outage probabilities, E-SSE, and energy efficiencies, showing that the RIS/IOS significantly enhances V2V communication performance. Table 1 compares the contributions of the proposed work compared to the existing works. The components considered are marked with a checkmark, while those not taken into account are marked with a cross.

### 2.6. Research Gaps

The extensive literature on NOMA confirms its suitability for massive connectivity in next-generation wireless networks. However, most existing studies focus on two-user NOMA systems, which limits their applicability to real-world multi-user scenarios. One of the major challenges in NOMA is iSIC, leading to significant error propagation, especially in multi-user environments. Very few studies examine the impact of realistic HWDs from gNB and UE. To address these issues, HNOMA has been introduced to minimize SIC errors and HWDs while reducing system complexity. Despite allocating higher power to the WUE, cell-edge users experience severe performance degradation in terms of outage probability, SSE, and throughput. C-NOMA enhances the performance of the WUE by allowing retransmission from the SUE and introducing diversity gain. However, this comes at a cost of increased energy consumption for the SUE, limiting system efficiency. To overcome this challenge, an RIS has been integrated into C-NOMA, enabling the SUE to retransmit with lower power consumption. However, RIS-based solutions are constrained by their half-space coverage, which restricts their ability to fully optimize signal propagation. IOSs offer a promising solution by enabling full-space coverage and ensuring efficient signal steering towards the intended UE. To the best of our knowledge, the integration of IOSs in C-HNOMA systems has not been examined in the existing literature. Addressing this gap, our work aims to investigate the potential of IOS-assisted C-HNOMA to improve system performance by enhancing retransmission efficiency and reducing coverage limitations.

### 2.7. Major Contributions

The key contributions of this work are summarized as follows:

1. The analytical expressions for outage probability in multi-user IOS-aided C-HNOMA systems are derived, considering various user pairing schemes.
2. A novel optimization framework is proposed to allocate power fractions dynamically within each time slot, maximizing the overall SSE while maintaining computational efficiency.
3. The complexity of different user pairing strategies is analyzed, and their effectiveness in enhancing system performance is systematically evaluated.
4. The analytical expressions for throughput and diversity gain are derived as a function of *L*, providing key insights into the system’s scalability and performance trade-offs.
5. The effects of iSIC and HWDs on outage probability, throughput, and SSE are thoroughly examined, providing a comprehensive understanding of system limitations and possible enhancements.

## 3. System Model

### 3.1. Framework for HNOMA

This section outlines the pairing schemes used in HNOMA systems. As mentioned, NOMA is a prominent technology supporting dense connection networks. However, the SIC inherent to NOMA can complicate the system design and degrade overall performance. HNOMA has been introduced as a combination of OMA and NOMA to address this issue. This approach benefits from the simplicity of the OMA structure while achieving the massive connectivity offered by NOMA systems. The system combines time division multiple access (TDMA) with the NOMA framework, specifically designed for *M* UE systems.

**Figure 1:**
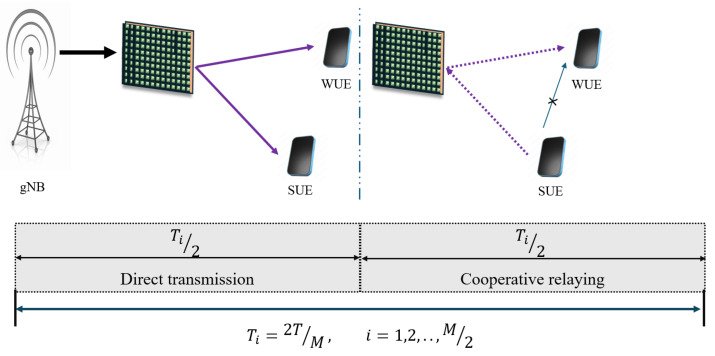
The sub-frame allocation for the IOS-aided C-HNOMA system.

Figure 1 illustrates the ith sub-frame allocated to the *p*th pairing scheme for the IOS-aided hybrid cooperation NOMA (C-HNOMA) system. The total time frame is denoted as *T*, which is divided into $T_{i}=2T/M$ sub-frames in a *M*-user system. In the *M*-user system, the total time frame, *T*, is divided into M/2 sub-frames. During each sub-frame, pairs of devices are allowed to have DL transmissions from gNB. Within each sub-sub-frame, the paired UE uses a two-UE C-NOMA system. This arrangement results in a two-user IOS-aided C-HNOMA system within the sub-frames. A crucial aspect of this setup is the process of pairing the UE. This section elaborates on the various pairing schemes and the complexities associated with each. Figure 1 pertains to the ith sub-frame. During $T_{i}/2$, there is a transmission from gNB to the SUE and WUE through IOSs. In the remaining $T_{i}/2$, cooperative relaying occurs between the SUE and WUE through IOSs. The direct link between the SUE and WUE during the cooperative relaying is assumed to have significant blockage. Hence, the effect of the direct link is negligible.

The following assumptions are established for this system: the gNB and all connected UE function using a single antenna architecture to simplify the overall design and signal processing. The total number of devices, represented as *M*, is in the power of 2, allowing two pieces of UE in each pair. Additionally, the devices are ordered based on their channel gains, which are dependent on the distance from the gNB to the path-loss exponent; the UE closest to the gNB is identified as the strongest, benefiting from maximum signal quality, while the farthest UE is the weakest, experiencing the poorest signal reception. This structured ordering is essential for optimizing the PA and network’s performance.

**Figure 2:**
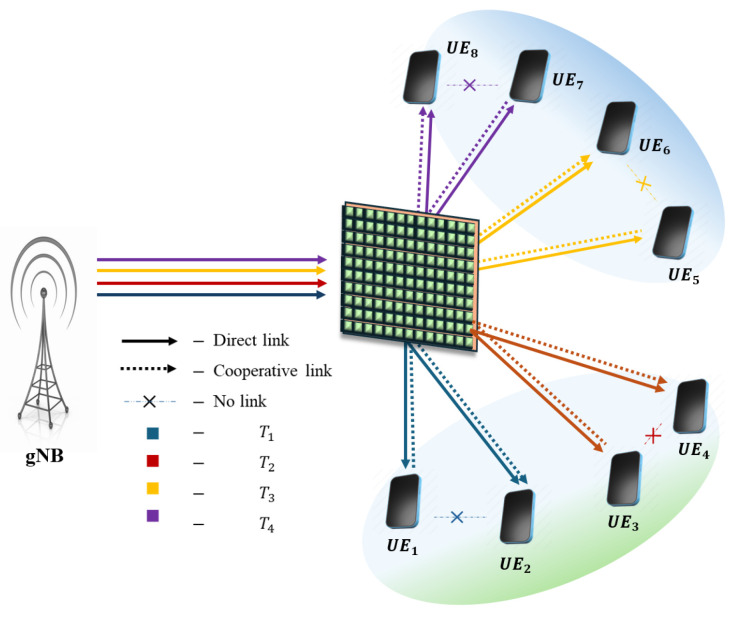
Schematic of the proposed IOS-aided C-HNOMA system with NN-FF pairing.

**Figure 3:**
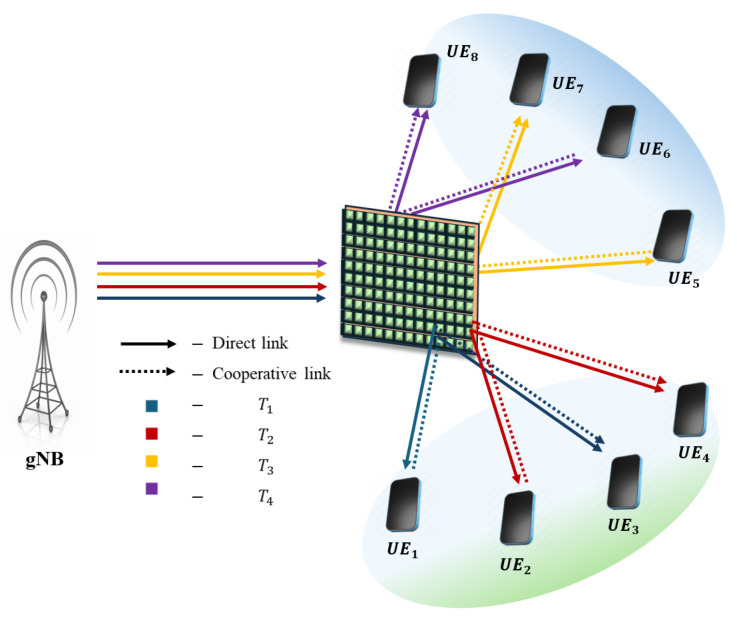
Schematic of the proposed IOS-aided C-HNOMA system with OO-EE pairing.

**Figure 4:**
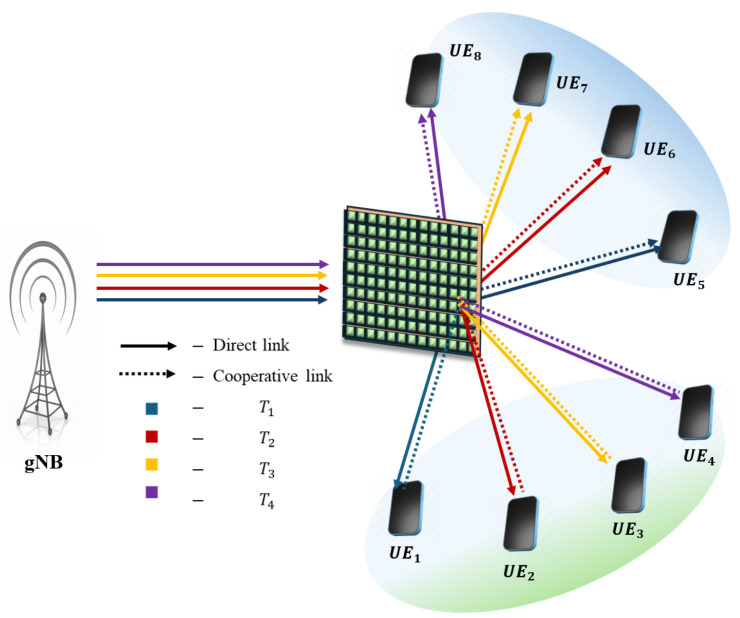
Schematic of the proposed IOS-aided C-HNOMA system with SW-SW pairing.

**Figure 5:**
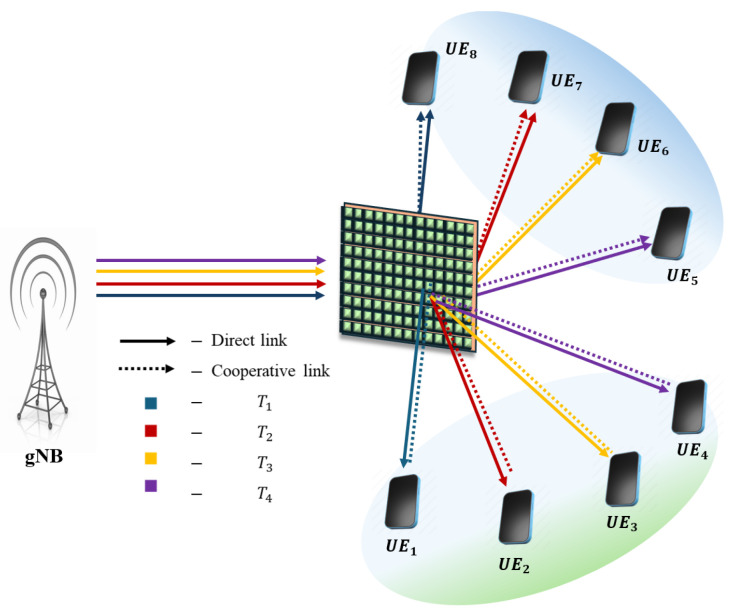
Schematic of the proposed IOS-aided C-HNOMA system with SW pairing.

**Figure 6:**
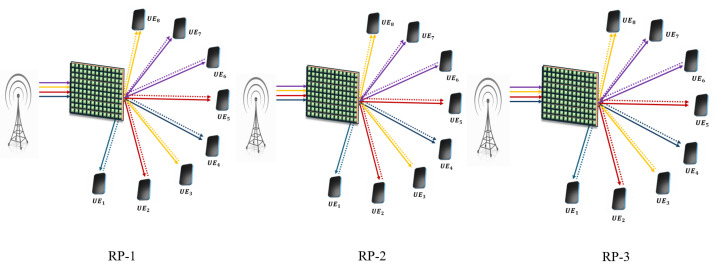
Schematic of the proposed IOS-aided C-HNOMA system with RP scheme.

**NOTE:** The manuscript states that the device with the higher channel gain in each pair is considered the SUE, while the other device is regarded as the WUE. For the description of pairing schemes, the pairing of eight UE/IoT devices with various pairing schemes is illustrated below. The assumption is that UE_1_, UE_2_, UE_3_, and UE_4_ are on the reflecting side of the IOS, and UE_5_, UE_6_, UE_7_, and UE_8_ are on the refracted side of the IOS. From Figure 2, Figure 3, Figure 4, Figure 5 and Figure 6, a DL transmission from gNB to UE through the IOS is presented. The total time frame is equally divided into four sub-frames for eight pieces of UE, represented as $T_{1}$, $T_{2}$, $T_{3}$, and $T_{4}$, as shown in blue, red, yellow, and purple, respectively, in the figures. The transmissions from gNB to UE through the IOS in the direct transmissions in the sub-frames are represented as solid lines in the figures from Figure 2, Figure 3, Figure 4, Figure 5 and Figure 6. As mentioned before, within each sub-frame, part of the time frame is allotted for direct transmission, while the remaining time frame is allocated for the SUE to relay the WUEs’ message during cooperative relaying. The cooperative links between the SUE and WUE are represented as dotted lines in Figure 2, Figure 3, Figure 4, Figure 5 and Figure 6. The paired UE in every sub-frame for various pairing schemes is listed in Table 2.

- **Near–near and far–far (NN-FF) pairing [27]:** Figure 2 illustrates NN-FF pairing. In this configuration, adjacent pieces of UE are paired, resulting in similar channel gains. In each sub-frame, gNB transmits the composite signals of UE_1_ and UE_2_ when i=1, UE_3_ and UE_4_ when i=2, UE_5_ and UE_6_ when i=3, and UE_7_ and UE_8_ when i=4. This kind of pairing experiences less complexity with the complexity order of O(M) [21,28].

- **Odd–odd and even–even (OO-EE) pairing:** In the context of NN-FF pairing, the pairs experience similar channel gains. This configuration is not conducive to the NOMA phenomenon. Consequently, to augment diversity in the channel gains of each pair, devices categorized as odd–odd and even–even are paired according to their respective channel gains. This methodology enhances system performance; however, it also elevates the complexity to a significant degree, resulting in $O\left(MlogM\right)$. The schematic representation for OO-EE pairing is delineated in Figure 3.

- **Stronger–weak strong–weak (SW-SW) pairing:** Figure 4 illustrates SW-SW pairing. The OO-EE pairing demands the value of *M* to be a power of 2 in contrast with other pairing schemes. To address this limitation, the devices are ordered according to their channel gains and divided into two equal groups. The first group consists of the first half of the SUE, and the next group contains the second half of the WUE. The devices within each group are chosen as a pair to ensure a uniform channel distribution in every pairing. This takes the complexity order of $O\left(MlogM\right)$ [28].

- **Strong–weak (SW) pairing:** The SW pairing method is examined, as illustrated in Figure 5. In this pairing approach, the strongest UE is paired with the weakest UE, followed by the next strongest UE being paired with the weakest UE, adhering to the complexity order of O(2M) [21,27].

- **Random pairing (RP):** In order to assess the effectiveness of the pairing schemes, three different RP schemes are highlighted. RP schemes are listed in Table 2, each outlining a unique set of pairs. Visual representations are shown in Figure 6. The different UE is randomly paired irrespective of channel gains or any rules. This RP results in O(MlogM) for the worst case and O(M) for the best case [21]. Table 3 provides the definitions for the symbols and notations used in this work.

In the context of C-HNOMA, each sub-frame is further divided into two equal slots. The first half is for direct transmission, while the second half employs cooperative decode-and-forward (DF) relaying. During the direct transmission, the composite signal for the *p*th pair at the *i*th time slot is transmitted. The power fraction allocated to the SUE during the *i*th time slot is denoted as $\mu_{si}$, while the power fraction allocated to the WUE is $\mu_{wi}$. To ensure fairness, it is required that $0\;<\mu_{si}\;\leq \;\mu_{wi}<1$ and that $\mu_{si}+\mu_{wi}=1$. The total transmit power budget is denoted as *P*. The composite signal transmitted for the *p*th pairing during the *i*th time slot in the direct transmission is given by the following:

(1) $$X_{p}^{i}=\left\{\begin{array}{cc} \begin{array}{c} \sqrt{P\mu_{si}}x_{2i-1}+\sqrt{P\mu_{wi}}x_{2i}\\ \left(\frac{1-\left({-1}^{i}\right)}{2}\right)\left[\sqrt{P\mu_{si}}x_{2i-1}+\sqrt{P\mu_{wi}}x_{2i+1}\right]+\left(\frac{1+\left({-1}^{i}\right)}{2}\right)\left[\sqrt{P\mu_{si}}x_{2i-2}+\sqrt{P\mu_{wi}}x_{2i}\right] \end{array}\; & \begin{array}{c} ;p=NN-FF\\ ;p=OO-EE \end{array}\\ \sqrt{P\mu_{si}}x_{2i-1}+\sqrt{P\mu_{wi}}x_{2i} & ;p=SW-SW\\ \begin{array}{c} \sqrt{P\mu_{si}}x_{i}+\sqrt{P\mu_{wi}}x_{M+1-i}\\ \sqrt{P\mu_{si}}x_{r_{1}}+\sqrt{P\mu_{wi}}x_{r_{2}};r_{1}\neq r_{2}\;\forall \;i,\left(x_{r_{1}},x_{r_{2}}\right)\in x_{u} \end{array} & \begin{array}{c} ;p=SW\;\\ ;p=RP\; \end{array} \end{array}\right.$$

### 3.2. Framework for UEs Under iSIC and HWDs

This section elaborates on the system model for a multi-user scenario, considering the iSIC and HWIs. The SNR achieved during the direct transmission and cooperative relaying of the IOS-aided C-HNOMA system is described. The received signal at UE during direct transmission is given by the following:

(2) $$y_{d-u}^{r,f}=h_{d}\Theta^{r,f}g_{d-u}+w_{u}+n_{u}$$

where ${\left[h_{d}\right]}_{L\times 1}$ is the channel between gNB and IOS. $h_{d}^{H}$ = $\left[\alpha_{1}e^{-j\theta_{1}},\alpha_{2}e^{-j\theta_{2}},\;\ldots ,{\;\alpha }_{L}e^{-j\theta_{L}}\right]$, $\alpha_{l}e^{-j\theta_{l}}∼CN\left(0,1\right),l=1,2\ldots L,\;h_{d}∼CN\left(0,1\right)$. The IOS phase shift matrix is ${\left[\Theta^{r,f}\right]}_{L\times L}=diag\left(\chi_{1}^{r,f}e^{j\phi_{1}^{r,f}},\;\;\chi_{2}^{r,f}e^{j\phi_{2}^{r,f}},\ldots ,\chi_{L}^{r,f}e^{j\phi_{L}^{r,f}}\right)$, $\theta_{l\;}\epsilon \left(0,2\pi \right]$. $\chi_{l}^{r,f}\epsilon \left(\chi_{l}^{r},\chi_{l}^{f}\right)$, $\chi_{l}^{r}\epsilon \left(0,1\right)$. The IOS operates in three modes: energy splitting (ES), mode switching (MS), and time splitting (TS) [14,15]. In this context, the MS protocol is preferred due to its simpler hardware complexity compared to the ES protocol. The entire time frame is utilized for reflection/refraction in the MS protocol, leading to more efficient spectrum use compared to the TS protocol [31]. $\chi_{l}^{r}=0$ for the UE under the refracting side of the IOS and $\chi_{l}^{r}=1$ for the UE under the reflecting side of the IOS. Similarly, $\chi_{l}^{f}\epsilon \left(0,1\right)$, $\chi_{l}^{f}=1$ for the UE under the refractive side of the IOS and $\chi_{l}^{f}=0$ for the UE under the reflecting side of the IOS. $n_{u}∼CN\left(0,\sigma_{u}^{2}\right)$ is the noise component added at the UE. It is assumed that $\sigma_{u}^{2}$ is common for all the UE, where $\sigma_{1}^{2}=\sigma_{2}^{2}=$…$=\sigma_{M}^{2}=\sigma_{u}^{2}$. $w_{u}$ is the hardware noise term $w_{u}\epsilon \left(w_{s},\;w_{w}\right)$. ${\left[g_{d-u}\right]}_{L\times 1}$ is the channel between the IOS and UE, which is given as follows: $g_{d-u}={\left[\begin{array}{c} \beta_{1}e^{-j\varphi_{1}},\;\;\beta_{2}e^{-j\varphi_{2}},\;\ldots ,\beta_{L}e^{-j\varphi_{L}} \end{array}\right]}^{H}$, where $\beta_{l}e^{-j\varphi_{l}}∼CN\left(0,\delta_{u}^{2}\right).\;$The phase compensation produced via the IOS is $\theta_{l}=-\psi_{l}-\varphi_{l}$. The cumulative channel between gNB and the UE is $H_{u}=h_{d}\Theta^{r,f}g_{d-u}$.

The SE achieved by the SUE during direct transmission is given by the following:

(3) $$\Gamma_{d-s,p}^{i}=\frac{1}{M}\log_{2}\left(1+\frac{G_{s}\rho \mu_{si}}{\varepsilon G_{s}\rho \mu_{wi}+G_{s}\rho \left[k_{g}^{2}+k_{s}^{2}\right]+1}\right)$$

where

(4) $$G_{s}=\left\{\begin{array}{cc} \begin{array}{c} {\left|H_{2i-1}\right|}^{2}\\ \left(\frac{1-\left({-1}^{i}\right)}{2}\right){\left|H_{2i-1}\right|}^{2}+\left(\frac{1+\left({-1}^{i}\right)}{2}\right){\left|H_{2i-2}\right|}^{2} \end{array}\; & \begin{array}{c} ;p=NN-FF\\ ;p=OO-EE \end{array}\\ {\left|H_{2i-1}\right|}^{2} & ;p=SW-SW\\ \begin{array}{c} {\left|H_{i}\right|}^{2}\\ {\left|H_{r_{1}}\right|}^{2} \end{array} & \begin{array}{c} ;p=SW\;\\ ;p=RP\; \end{array} \end{array}\right.\;;i=1,2,\ldots ,\;M/2$$

$\rho =\frac{P}{\sigma_{u}^{2}}$ is the instantaneous SNR. The SE achieved by the WUE during direct transmission is given by the following:

(5) $$\Gamma_{d-w,p}^{i}=\frac{1}{M}\log_{2}\left(1+\frac{G_{w}\rho \mu_{wi}}{G_{w}\rho \mu_{si}+G_{w}\rho \left[k_{g}^{2}+k_{w}^{2}\right]+1}\right)$$

where

(6) $$G_{w}=\left\{\begin{array}{cc} \begin{array}{c} {\left|H_{2i}\right|}^{2}\\ \left(\frac{1-\left({-1}^{i}\right)}{2}\right){\left|H_{2i+1}\right|}^{2}+\left(\frac{1+\left({-1}^{i}\right)}{2}\right){\left|H_{2i}\right|}^{2} \end{array} & \begin{array}{c} ;p=NN-FF\\ ;p=OO-EE \end{array}\\ {\left|H_{2i}\right|}^{2} & ;p=SW-SW\\ \begin{array}{c} {\left|H_{M+1-i}\right|}^{2}\\ {\left|H_{r_{2}}\right|}^{2} \end{array} & \begin{array}{c} ;p=SW\;\\ ;p=RP\; \end{array} \end{array}\right.\;;i=1,2,\ldots ,\;M/2$$

Here, ${\left|H_{u}\right|}^{2}$ is the cumulative channel gain of the *u*th UE, where ${\left|H_{u}\right|}^{2}\;\epsilon \left\{G_{s},G_{w}\right\}$. The SE achieved by the WUE at the SUE signal during direct transmission is given by [32] the following:

(7) $$\Gamma_{d-ws,p}^{i}=\frac{1}{M}\log_{2}\left(1+\frac{G_{s}\rho \mu_{wi}}{G_{s}\rho \mu_{si}+G_{s}\rho \left[k_{g}^{2}+k_{s}^{2}\right]+1}\right)$$

Using the NOMA phenomenon, the SUE must decode the WUE message signal to perform SIC. During the cooperative relaying, the strong UE retransmits a decoded copy of the WUE message to the WUE. The received signal at the WUE at *p*th pairing and *i*th sub-frame during cooperative relaying is given by [9]

(8) $$y_{c-u}^{r,f}=h_{c-u}\Theta^{r,f}g_{c-u}+w_{u}+n_{u}$$

where ${\left[h_{c-u}\;\right]}_{L\times 1}$ is the channel between the SUE and IOS. ${\left[g_{c-u}\right]}_{L\times 1}$ is the channel between the IOS and WUE. The SE achieved by the WUE during cooperative relaying is given by [9,32]

(9) $$\Gamma_{c-w,p}^{i}=\frac{1}{M}\log_{2}\left(1+\frac{G_{c-p}\rho }{G_{c-p}\rho \left[k_{s}^{2}+k_{w}^{2}\right]+1}\right)$$

$G_{c-p}$ is the channel between the SUE and WUE during cooperative relaying, which is given by

(10) $$G_{c-p}={\left|H_{s,w}\right|}^{2}=\left\{\begin{array}{cc} \begin{array}{c} {\left|H_{2i-1,2i}\right|}^{2}\\ \left(\frac{1-\left({-1}^{i}\right)}{2}\right){\left|H_{2i-1,2i+1}\right|}^{2}+\left(\frac{1+\left({-1}^{i}\right)}{2}\right){\left|H_{2i-2,2i}\right|}^{2} \end{array}\; & \begin{array}{c} ;p=NN-FF\\ ;p=OO-EE \end{array}\\ {\left|H_{2i-1,2i}\right|}^{2} & ;p=SW-SW\\ \begin{array}{c} {\left|H_{i,M+1-i}\right|}^{2}\\ {\left|H_{r_{1},r_{2}}\right|}^{2} \end{array} & \begin{array}{c} ;p=SW\;\\ ;p=RP\; \end{array} \end{array}\right.\;;i=1,2,\ldots ,\;M/2$$

## 4. Performance Analysis

This section presents detailed derivations and closed-form expressions for the probability of outage, diversity order, and throughput of the proposed IOS-aided C-HNOMA system for multiple users. Additionally, it includes the optimization of power fraction to maximize SSE under the constraints of iSIC and HWDs.

### 4.1. Outage Probability

The average probability of outage of the SUE for a frame duration *T* is calculated through the condition $\Gamma_{d-ws,p}^{i}<{\tilde{R}}_{w,p}^{i}\cup \Gamma_{d-s,p}^{i}<{\tilde{R}}_{s,p}^{i}$, where ${\tilde{R}}_{w,p}^{i}$ and ${\tilde{R}}_{s,p}^{i}$ are the minimum SE requirements of the WUE and SUE, respectively. ${\tilde{R}}_{w,p}^{i},{\tilde{R}}_{s,p}^{i}\;\epsilon \;{\tilde{R}}_{u}$. ${\tilde{R}}_{u}$ is the minimum SE requirement of the *u*th UE. The probability of outage at the SUE during direct transmission is $P_{s,p}^{i}=P\left(\Gamma_{d-ws,p}^{i}<{\tilde{R}}_{w,p}^{i},\Gamma_{d-s,p}^{i}<{\tilde{R}}_{s,p}^{i}\right)$. The channel condition $H_{u}=H_{s,p}^{i}$ for the SUE experiencing outage is given by

(11) $$P_{s,p}^{i}=P\left(H_{s,p}^{i}<\sqrt{\frac{\rho_{w,p}^{i}}{\rho \mu_{wi}}}\sqrt{\frac{\mu_{wi}}{\rho_{w,p}^{i}\left(\mu_{si}+\left[k_{g}^{2}+k_{w}^{2}\right]\right)}}\right)\times P\left(H_{s,p}^{i}<\sqrt{\frac{\rho_{s,p}^{i}}{\rho \mu_{si}}}\sqrt{\frac{\mu_{si}}{\rho_{s,p}^{i}\left(\varepsilon \mu_{wi}+\left[k_{g}^{2}+k_{w}^{2}\right]\right)}}\right)$$

where $\rho_{s,p}^{i}$ and $\rho_{w,p}^{i}$ are the SNR thresholds for the SUE and WUE. The relation for the SNR threshold and minimum SE requirement is given as $\rho_{s,p}^{i}=2^{M{\tilde{R}}_{s,p}^{i}/2}-1$ and $\rho_{w,p}^{i}=2^{M{\tilde{R}}_{w,p}^{i}/2}-1$. For a detailed derivation of (11), refer to Appendix A. The probability density function (PDF) for the random variable $H_{s,p}^{t}\;$is expressed as [33]

(12) $$f_{H_{s,p}^{i}}\left(h_{s,p}^{i}\right)=\frac{{h_{s,p}^{i}}^{a}}{b^{a+1}\Gamma \left(a+1\right)}e^{-\left(h_{s,p}^{i}/b\right)}dh_{s,p}^{i}$$

where $a\;=\frac{{\left(m_{s,p}^{i}\right)}^{2}}{{\sigma_{s,p}^{i}}^{2}}-1$ and $b=\frac{{\sigma_{s,p}^{i}}^{2}}{m_{s,p}^{i}}$[33,34] with a mean of $m_{s,p}^{i}=\frac{L_{s,p}^{i}\pi }{2}$and variance of ${\sigma_{s,p}^{i}}^{2}=L_{s,p}^{i}\left(1-\frac{\pi^{2}}{16}\right)$[32]. Here, $L_{s,p}^{i}$ is given by

(13) $$L_{s,p}^{i}=\left\{\begin{array}{cc} \begin{array}{c} L_{2i-1}\\ \left(\frac{1-\left({-1}^{i}\right)}{2}\right)L_{2i-1}+\left(\frac{1+\left({-1}^{i}\right)}{2}\right)L_{2i-2} \end{array}\; & \begin{array}{c} ;p=NN-FF\\ ;p=OO-EE \end{array}\\ L_{2i-1} & ;p=SW-SW\\ \begin{array}{c} L_{i}\\ L_{r_{1}} \end{array}\; & \begin{array}{c} \;;p=SW\;\;\;\;\;\;\;\;\;\;\;\;\\ \;;p=RP\;\;\;\;\;\;\;\;\;\;\;\;\; \end{array} \end{array}\right.$$

$L_{s,p}^{i}$ is the number of IOS components allocated to the SUE during the *i*th sub-frame and *p*th pairing. By substituting the PDF of $H_{s,p}^{i}$ and solving as in [29,34], the outage probability at the SUE during direct transmission is determined as

(14) $$P_{s,p}^{i}=\left(\frac{\gamma \left(a+1,\frac{x_{1}}{b}\right)}{\Gamma \left(a+1\right)}\right)\times \left(\frac{\gamma \left(a+1,\frac{x_{2}}{b}\right)}{\Gamma \left(a+1\right)}\right)$$

where $x_{1}=\sqrt{\frac{\mu_{wi}}{\rho_{w,p}^{i}\left(\mu_{si}+\left[k_{g}^{2}+k_{w}^{2}\right]\right)}}\sqrt{\frac{\rho_{w,p}^{i}}{\rho \mu_{wi}}}$ and $x_{2}=\sqrt{\frac{\mu_{si}}{\rho_{s,p}^{i}\left(\varepsilon \mu_{wi}+\left[k_{g}^{2}+k_{w}^{2}\right]\right)}}\sqrt{\frac{\rho_{s,p}^{i}}{\rho \mu_{si}}}$. Substituting $a,\;b,\;x_{1}$ and $x_{2}$ in (14) gives

(15) $$P_{s,p}^{i}=\left(\frac{\gamma \left(\frac{\pi^{2}L_{s,p}^{i}}{16-\pi^{2}},\frac{2\pi }{16-\pi^{2}}\sqrt{\frac{\mu_{wi}}{\rho_{w,p}^{i}\left(\mu_{si}+\left[k_{g}^{2}+k_{w}^{2}\right]\right)}}\sqrt{\frac{\rho_{w,p}^{i}}{\rho \mu_{wi}}}\right)}{\Gamma \left(\frac{\pi^{2}L_{s,p}^{i}}{16-\pi^{2}}\right)}\right)\times \left(\frac{\gamma \left(\frac{\pi^{2}L_{s,p}^{i}}{16-\pi^{2}},\frac{2\pi }{16-\pi^{2}}\sqrt{\frac{\mu_{si}}{\rho_{s,p}^{i}\left(\varepsilon \mu_{wi}+\left[k_{g}^{2}+k_{w}^{2}\right]\right)}}\sqrt{\frac{\rho_{s,p}^{i}}{\rho \mu_{si}}}\right)}{\Gamma \left(\frac{\pi^{2}L_{s,p}^{i}}{16-\pi^{2}}\right)}\right)$$

The condition for the WUE experiencing outage is $\Gamma_{d-w,p}^{i}<{\tilde{R}}_{w,p}^{i}$. The probability of outage at the WUE during direct transmission is $P_{w,p}^{i}=P\left(\Gamma_{d-w,p}^{i}<{\tilde{R}}_{w,p}^{i}\right)$. Rewriting with respect to the channel condition $H_{u}=H_{w,p}^{i}$, the condition for the WUE experiencing outage is given by

(16) $$P_{w,p}^{i}=P\left(H_{w,p}^{i}<\sqrt{\frac{\mu_{wi}}{\rho_{w,p}^{i}\left(\mu_{si}+\left[k_{g}^{2}+k_{w}^{2}\right]\right)}}\sqrt{\frac{\rho_{w,p}^{i}}{\rho \mu_{wi}}}\right)$$

For a detailed derivation of (16), refer to Appendix B. Similarly, the PDF for $H_{w,p}^{i}\;$is expressed as [33]

(17) $$f_{H_{w,p}^{i}}\left(h_{w,p}^{i}\right)=\frac{{h_{w,p}^{i}}^{c}}{d^{c+1}\Gamma \left(c+1\right)}e^{-\left(h_{w,p}^{i}/d\right)}dh_{w,p}^{i}$$

where $c\;=\frac{{\left(m_{w,p}^{i}\right)}^{2}}{{\sigma_{w,p}^{i}}^{2}}-1$ and $d=\frac{{\sigma_{w,p}^{i}}^{2}}{m_{w,p}^{i}}$[33,34] with a mean of $m_{w,p}^{i}=\frac{L_{w,p}^{i}\pi }{2}$ and variance of ${\sigma_{w,p}^{i}}^{2}=L_{w,p}^{i}\left(1-\frac{\pi^{2}}{16}\right)$[32]. Here, $L_{w,p}^{i}$ is given by

(18) $$L_{w,p}^{i}=\left\{\begin{array}{cc} \begin{array}{c} L_{2i}\\ \left(\frac{1-\left({-1}^{i}\right)}{2}\right)L_{2i+1}+\left(\frac{1+\left({-1}^{i}\right)}{2}\right)L_{2i} \end{array}\; & \begin{array}{c} ;p=NN-FF\\ ;p=OO-EE \end{array}\\ L_{2i} & ;p=SW-SW\\ \begin{array}{c} L_{M+1-i}\\ L_{r_{2}} \end{array} & \begin{array}{c} ;p=SW\;\\ ;p=RP\; \end{array} \end{array}\right.$$

$L_{w,p}^{i}$ is the number of IOS components allocated to the WUE during the *i*th sub-frame and *p*th pairing. By substituting the PDF of $H_{w,p}^{i}$ and solving as in [29,34], the outage probability at the WUE during direct transmission is determined as

(19) $$P_{w,p}^{i}=\left(\frac{\gamma \left(c+1,\frac{x_{3}}{d}\right)}{\Gamma \left(c+1\right)}\right)$$

where $x_{3}=\sqrt{\frac{\mu_{wi}}{\rho_{w,p}^{i}\left(\mu_{si}+\left[k_{g}^{2}+k_{w}^{2}\right]\right)}}\sqrt{\frac{\rho_{w,p}^{i}}{\rho \mu_{wi}}}\;$. Substituting c,d, and $x_{3}$ in (19) gives

(20) $$P_{w,p}^{i}=\frac{\gamma \left(\frac{\pi^{2}L_{w,p}^{i}}{16-\pi^{2}},\frac{2\pi }{16-\pi^{2}}\sqrt{\frac{\mu_{wi}}{\rho_{w,p}^{i}\left(\mu_{si}+\left[k_{g}^{2}+k_{w}^{2}\right]\right)}}\sqrt{\frac{\rho_{w,p}^{i}}{\rho \mu_{wi}}}\right)}{\Gamma \left(\frac{\pi^{2}L_{w,p}^{i}}{16-\pi^{2}}\right)}$$

The condition for outage at the WUE during cooperative relaying is $\Gamma_{c-w,p}^{i}<{\tilde{R}}_{w,p}^{i}$. The probability of outage at the WUE during cooperative relaying is $P_{c-w,p}^{i}=P\left(\Gamma_{c-w,p}^{i}<{\tilde{R}}_{w,p}^{i}\right)$. Rewriting with respect to $H_{c-w,p}^{i}$, the condition for the WUE experiencing outage during cooperative relaying can be expressed as follows:

(21) $$P_{c-w,p}^{i}=P\left(H_{c-w,p}^{i}<\sqrt{\frac{1}{\rho_{w,p}^{i}\left(k_{g}^{2}+k_{w}^{2}\right)}}\sqrt{\frac{\rho_{w,p}^{i}}{\rho }}\right)$$

For a detailed derivation of (21), refer to Appendix C. Similarly, the PDF for the random variable $H_{c-w,p}^{i}\;$is expressed as [33]

(22) $$f_{H_{c-w,p}^{i}}\left(h_{c-w,p}^{i}\right)=\frac{{h_{c-w,p}^{i}}^{e}}{f^{e+1}\Gamma \left(e+1\right)}e^{-\left(h_{c-w,p}^{i}/f\right)}dh_{c-w,p}^{i}$$

where $e\;=\frac{{\left(m_{c-w,p}^{i}\right)}^{2}}{{\sigma_{c-w,p}^{i}}^{2}}-1$ and $f=\frac{{\sigma_{c-w,p}^{i}}^{2}}{m_{c-w,p}^{i}}$, with a mean of $m_{c-w,p}^{i}=\frac{L\pi }{2}$ and variance of ${\sigma_{c-w,p}^{i}}^{2}=L\left(1-\frac{\pi^{2}}{16}\right)$[32]. By substituting the PDF of $H_{c-w,p}^{i}$ and solving as in [29,33], the outage probability of the WUE during cooperative relaying is determined as

(23) $$P_{c-w,p}^{i}=\left(\frac{\gamma \left(e+1,\frac{x_{4}}{f}\right)}{\Gamma \left(e+1\right)}\right)$$

where $x_{4}=\sqrt{\frac{1}{\rho_{w,p}^{i}\left(k_{g}^{2}+k_{w}^{2}\right)}}\sqrt{\frac{\rho_{w,p}^{i}}{\rho }}$. Substituting e,f, and $x_{4}$ in (23) gives

(24) $$P_{c-w,p}^{i}=\frac{\gamma \left(\frac{\pi^{2}L}{16-\pi^{2}},\frac{2\pi }{16-\pi^{2}}\sqrt{\frac{1}{\rho_{w,p}^{i}\left(k_{g}^{2}+k_{w}^{2}\right)}}\sqrt{\frac{\rho_{w,p}^{i}}{\rho }}.\;\right)}{\Gamma \left(\frac{\pi^{2}L}{16-\pi^{2}}\right)}$$

Using selection diversity, the WUE processes the signal received with minimum outage, which is given by

(25) $$P_{w}^{i}=min\left\{P_{w,p}^{i},\;P_{c-w,p}^{i}\right\}$$

To evaluate the overall performance of the system, the average outage probability of all of the WUE for a frame duration *T* is estimated using

(26) $$P_{w}=\frac{2}{M}\sum_{i=1}^{M/2}P_{w}^{i}=\frac{2}{M}\sum_{i=1}^{M/2}min\left\{P_{w,p}^{i},\;P_{c-w,p}^{i}\right\}$$

The outage probability of the SUE is dependent only on the direct transmission. Therefore, the average outage probability of the SUE for a frame duration *T* is estimated using

(27) $$P_{s}=\frac{2}{M}\sum_{i=1}^{M/2}P_{s,p}^{i}$$

The average throughput of the proposed system can be calculated using [14]

(28) $$T={\tilde{R}}_{s,p}^{i}\left(1-P_{s}\right)+{\tilde{R}}_{w,p}^{i}\;\left(1-P_{w}\right)$$

### 4.2. Diversity Order

Diversity order provides how fast the outage curves fall as *L* increases in the probability of outage. In order to offer insights into the derived outage probability expressions, the diversity order for both the SUE and WUE is determined using [29] as follows:

(29) $$D_{s}=-\underset{\rho \to \infty }{lim}\left(\frac{\log_{2}P_{s}\;}{{log}_{2}\rho \;}\right)\;$$

(30) $$D_{w}=-\underset{\rho \to \infty }{lim}\left(\frac{\log_{2}P_{w}\;}{{log}_{2}\rho \;}\right)\;$$

Substituting (27) in (29) and (26) in (30) gives

(31) $$D_{s}=\frac{L^{2}}{4}\left(\frac{\pi^{4}}{{\left(16-\pi^{2}\right)}^{2}}\right)$$

(32) $$D_{w}=min\left\{\frac{1}{2}\frac{\pi^{2}L}{16-\pi^{2}},\frac{1}{1.386}\frac{\pi^{2}L}{16-\pi^{2}}\right\}$$

### 4.3. Maximum SSE Through Power Fraction Optimization

The SSE measures the overall capacity of the system and facilitates performance evaluation. The SSE is calculated as the sum of the SE of the individual pieces of UE within the system. PA factors can be optimized for maximizing SSE by dynamically distributing power among the UE based on their channel conditions during direct transmission. In the case of cooperative relaying, there is no power splitting and, therefore, no need for PA optimization. The SSE is given by

(33) $$C_{p}^{i}=\Gamma_{d-s,p}^{i}+max\left\{\Gamma_{d-w,p}^{i}\;,\;\Gamma_{c-w,p}^{i}\right\}$$

Providing optimal power for the UE during direct transmission is crucial, particularly for the SUE, as they need to manage a complex SIC process and engage in cooperative communication. To ensure fairness, the SUE receives a lower power fraction. Furthermore, while decoding, the SUE must meet the QoS requirements of the WUE. Consequently, power fractions should be optimized to maximize SSE. The condition for the UE to achieve maximum SSE while satisfying the individual QoS requirements of each piece of UE is outlined as follows [4]:

(34) $$\max_{\mu_{si},\;\mu_{wi}}C_{p}^{i}\;$$

Subject to

$$\Gamma_{d-w,p}^{i}\geq {\tilde{R}}_{w,p}^{i}$$

$$\Gamma_{d-s,p}^{i}\geq {\tilde{R}}_{s,p}^{i}$$

$$\mu_{si}+\;\mu_{wi}=1$$

$$0\;<\mu_{si}\;\leq \;\mu_{wi}<1$$

The condition for the SUE with no outage is given by $\Gamma_{d-s,p}^{i}\geq {\tilde{R}}_{s,p}^{i}$. Substituting (3) and solving for $\mu_{si}$ gives

(35) $$\mu_{si}\geq A_{s,p}^{min}=\frac{\rho_{s,p}^{i}\left(\left(\varepsilon +\left[k_{g}^{2}+k_{s}^{2}\right]\right)G_{s}\rho +1\right)}{\left(\varepsilon \rho_{s,p}^{i}+1\right)G_{s}\rho }$$

where $\rho_{s,p}^{i}$is the threshold SNR of the SUE. The condition for the WUE experiencing no outage is $\Gamma_{d-w,p}^{i}\geq {\tilde{R}}_{w,p}^{i}$. Solving this condition gives

(36) $$\mu_{si}\leq A_{s,p}^{max}=\frac{G_{w}\rho -\rho_{w,p}^{i}\left(G_{w}\rho \left(\left[k_{g}^{2}+k_{w}^{2}\right]+1\right)\right)}{G_{w}\rho \left(\rho_{w,p}^{i}+1\right)}$$

where $\rho_{w,p}^{i}$is threshold SNR of the WUE. Combining (35) and (36) gives,

(37) $$A_{s,p}^{min}\leq \mu_{si}\leq A_{s,p}^{max}$$

In this optimization problem, both paired pieces of UE must meet their minimum QoS requirements while maximizing the SSE. In a NOMA system, the WUE is generally assigned the majority of the power, as the SUE benefits from better channel conditions and thus requires less power. However, this PA strategy may fail to satisfy the QoS requirements of both pieces of UE. To ensure that the SUE meets its minimum QoS requirements, the upper limit, as formulated in (37), is allocated accordingly.

(38) $$\mu_{si}^{opt}=A_{s,p}^{max}$$

As $\mu_{si}+\;\mu_{wi}=1$, the remaining power is allocated to the WUE, which is given by

(39) $$\mu_{wi}^{opt}=1-\mu_{si}^{opt}$$

The feasibility region for the optimal power fractions allocated for the SUE and the WUE can be obtained by the condition

(40) $$A_{s,p}^{min}\leq A_{s,p}^{max}$$

From (40), the minimum SNR requirement is given by

(41) $$\rho \geq \frac{\rho_{s,p}^{i}G_{w}\left(\rho_{w,p}^{i}+1\right)+\rho_{w,p}^{i}G_{s}\left(\rho_{s,p}^{i}+1\right)}{G_{w}G_{s}\left(1-\left(\varepsilon +\left[k_{g}^{2}+k_{w}^{2}\right]\right)\rho_{s,p}^{i}\rho_{w,p}^{i}-\left[k_{g}^{2}+k_{s}^{2}\right]\left(\rho_{s,p}^{i}+\rho_{w,p}^{i}+\rho_{s,p}^{i}\rho_{w,p}^{i}\varepsilon \right)\right)}$$

## 5. Results and Discussion

This section validates the derived analytical expressions for the outage probability of the SUE and WUE, throughput, and SSE. It examines the proposed system’s performance under iSIC and HWD conditions. The software used is MATLAB 2023a, and the parameters and the values used for simulation are tabulated in Table 4. The proposed IOS-aided C-HNOMA acts as the base system. It is integrated with existing pairing schemes [21,27,28] and traditional PA optimization techniques [4,22], and a comparison between them is conducted to highlight the importance of pairing and PA optimization in improving system performance.

**Figure 7:**
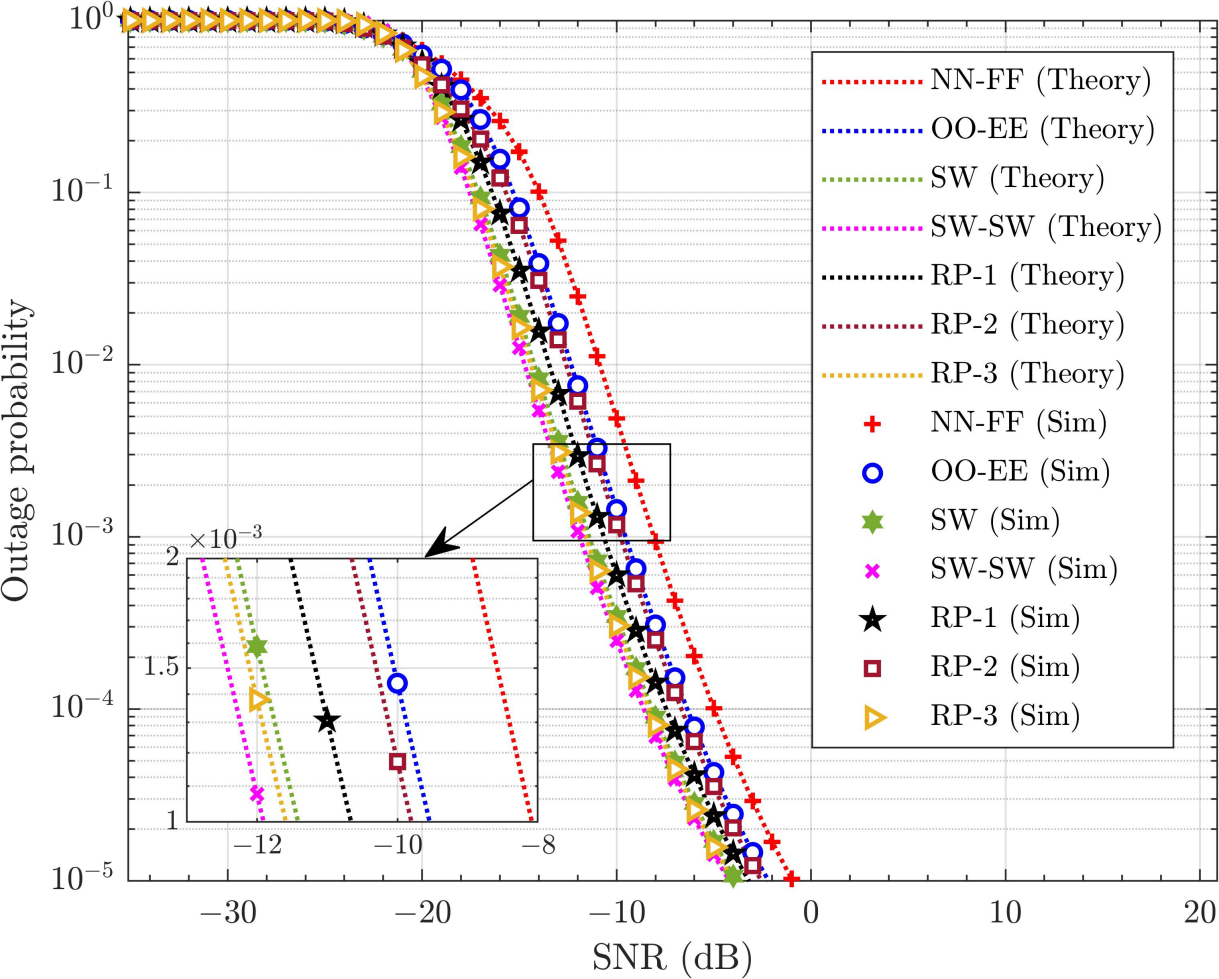
Average probability of outages for different pairing schemes of the SUE under ideal SIC and without HWD.

**Figure 8:**
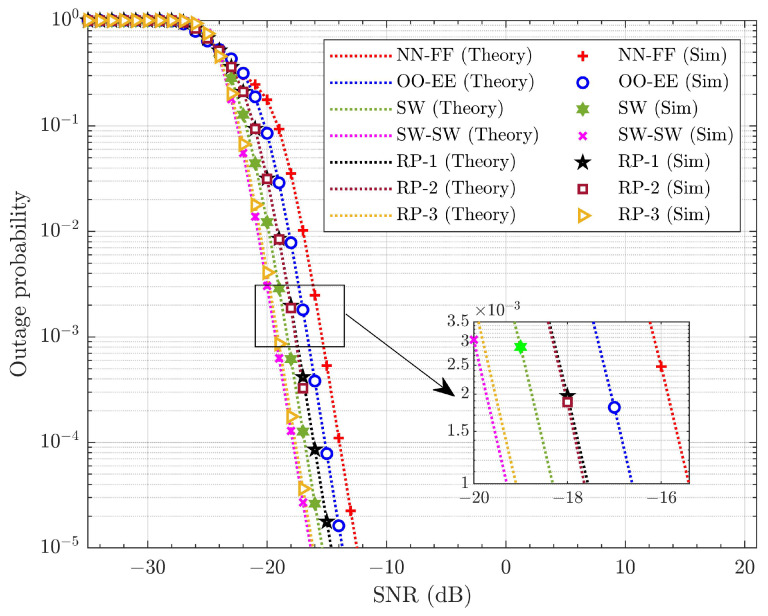
Average probability of outages for different pairing schemes of the WUE under ideal SIC and without HWD.

The probability of outage is the metric that defines the reliability of the wireless connections in the system. It is the ratio of the number of transmissions under outage to the total number of transmissions. Figure 7 illustrates the average outage performance of the SUE across different pairing schemes considering no SIC error or HWDs. The derived closed-form expressions for the average outage probability of the WUE and SUE are presented as seen in (26) and (27), respectively, and closely align with the simulation results. This confirms the accuracy of the derived average outage probability expressions. The probability of outage with SW-SW pairing shows improvement over other pairing strategies. Similarly, Figure 8 demonstrates the effectiveness of SW-SW pairing in the average outage probability performance of the WUE. The average probability of outage for the WUE is better than for the SUE. This improvement is attributed to the cooperative communication within each pair and high PA to the WUE. The average SNR gain observed by the SW-SW scheme over other pairing schemes for the SUE and WUE is tabulated in Table 5.

Table 5 clearly indicates that the SW-SW pairing enhances the outage performance in the SUE and WUE compared to other pairing schemes. This improvement arises from the SW-SW pairing’s provision of diverse channel gains within each pair. In contrast, the other pairing schemes exhibit similar channel gains, or some pairs in the system have similar channel gains while others demonstrate diverse channel gains, which ultimately diminishes the overall system performance. In terms of the SUE, SW-SW pairing offers ∼0.3 to ∼3.81 dB SNR gain, while for the WUE, it provides around ∼0.21 to ∼3.89 dB SNR gain. Three distinct cases of RP are presented here to evaluate the effectiveness of the pairing schemes. In RP, the UE is paired randomly, independent of channel gains or specific rules. As a result, they may not deliver consistent performance across various channel conditions. From Figure 7 and Figure 8, it is evident that SW-SW pairing has significant improvement over other pairing schemes. This represents a significant improvement, highlighting the importance of pairing within the system.

**Figure 9:**
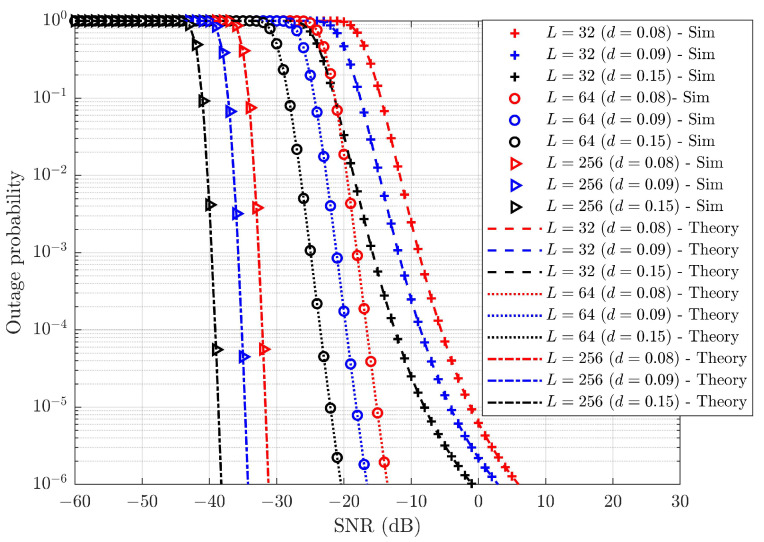
Average probability of outage of the SW-SW SUE under iSIC, ε=0.01, and HWDs for varying *L*.

**Figure 10:**
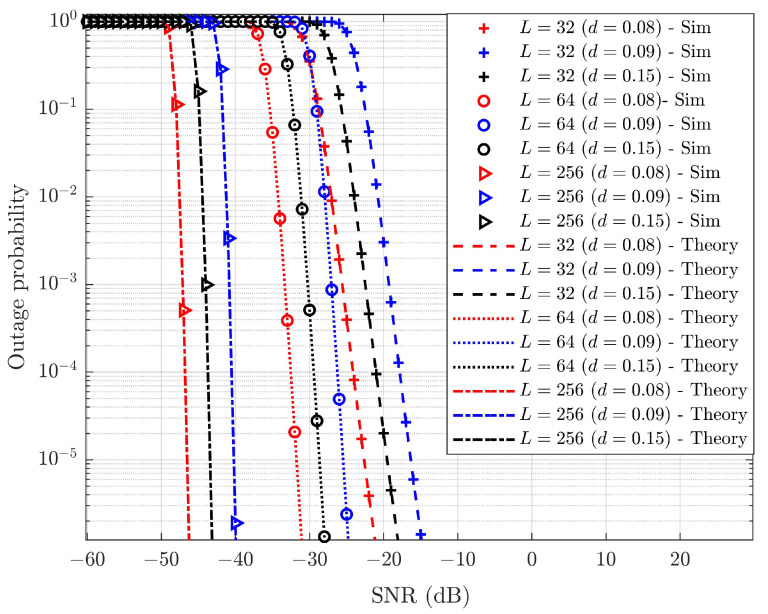
Average probability of outage of the SW-SW WUE under iSIC, ε=0.01, and HWDs for varying *L*.

In real-time scenarios, HWD and SIC errors arise. Considering iSIC and HWD conditions, the performance of the average outage probability of SW-SW pairing is evaluated. SIC error, ε=0.01, is fixed, and varying values of HWD conditions for the SUE and WUE have been evaluated, as shown in Figure 9 and Figure 10, respectively. The figures reveal that with HWDs, there is a considerable degradation in the outage probabilities for both the SUE and WUE. As *L* increases, the outage performance of the SUE and WUE improves even under iSIC and HWD conditions. It is also noted that as *L* increases, the target outage falls into the negative SNR region. Table 6 compares the outage probabilities of the SUE and WUE under iSIC and HWD conditions for different values of *L*.

Table 6 shows that the performance of the WUE in terms of outage is better than that of the SUE. This is due to only a small fraction of power being allocated to the SUE, which must perform the complex SIC process. Eventually, there exists iSIC in real-time systems, which consumes more power to reach the target outage. In contrast, the WUE is allocated a larger fraction of power, although the equipment may suffer from blockage and weak signal reception. By using cooperative relaying for the WUE, the outage performance of the WUE surpasses that of the SUE. From Table 6, HWD conditions in the system affect the average outage performance of both the SUE and WUE. An increase in HWD raises the SNR requirements needed to achieve the target outage probability. These constraints due to iSIC and HWDs can be mitigated by increasingL in the system. With 1% iSIC and 15% HWD, the SUE can achieve ∼16.55 to ∼30.49 dB SNR gains with L=256 compared to L=64 and 32 to reach an average outage probability of $10^{-5}$. For the WUE with 15% HWD, ∼14.74 to ∼23.87 dB of SNR gain is achieved with *L* = 256 compared toL = 64 and 32 to reach the target outage.

**Figure 11:**
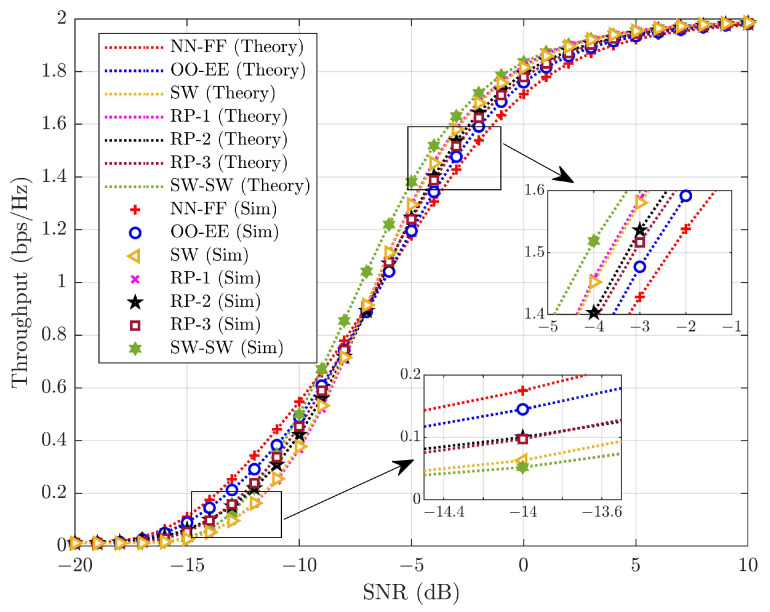
Throughput performance comparison under perfect SIC with no HWD across various pairing schemes at L=16.

Throughput is evaluated in Figure 11 for various pairing schemes with L=16 under ideal conditions (ε=0andd=0). From Figure 11, the expression for throughput, as in (28), exactly matches the simulations. ${\tilde{R}}_{s,p}^{i}=1$ and ${\tilde{R}}_{w,p}^{i}=1$ are established for throughput analysis, with throughput achieving 2 bps/Hz at a high SNR. It is observed that at a low SNR, the performance of NN-FF pairing dominates over other schemes. As the SNR increases, SW-SW pairing shows improved performance. This illustrates the trade-off between computational complexity and performance. Increased computational complexity necessitates more processing power and time to execute pairing, resulting in higher SNR requirements. Based on the SNR region, appropriate pairing can be applied. At an SNR of −15 dB, NN-FF, OO-EE, SW, RP-1, RP-2, RP-3, and SW-SW achieve throughputs of ∼0.111, ∼0.089, ∼0.063, ∼0.054, ∼0.030, ∼0.027, and ∼0.026 bps/Hz, respectively. Here, NN-FF shows a difference of ∼0.022 to ∼0.085 bps/Hz in throughput over other pairing schemes. NN-FF pairing demonstrates improved performance at an SNR of −15 dB because it has the lowest complexity order compared to other pairing schemes. However, for an SNR of −2 dB, NN-FF, OO-EE, SW, RP-1, RP-2, RP-3, and SW-SW achieve throughputs of ∼1.538, ∼1.592, ∼1.624, ∼1.644, ∼1.681, ∼1.687, and ∼1.719 bps/Hz, respectively. At a −2 dB SNR, SW-SW provides ∼0.032 to ∼0.181 bps/Hz difference in throughput over other pairing schemes. From Figure 11, it can be inferred that based on the application and requirement, a less complex NN-FF pairing can be chosen where performance is compromised, while for performance-oriented applications, SW-SW pairing can be applied, which may incur higher power consumption due to computational complexity.

**Figure 12:**
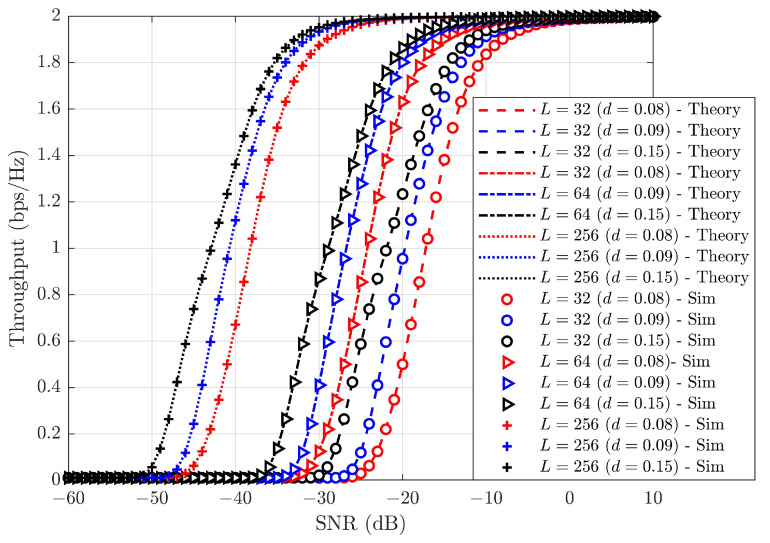
Throughput performance of SW-SW under varyingL with ε=0.01 and varying HWDs.

Figure 12 illustrates the throughput performance of SW-SW pairing under various *L* values while increasing HWDs under a fixed SIC error of 0.01. From Figure 12, it is apparent that throughput performance degrades as HWD increases. An increase in *L* results in enhanced throughput performance. Table 7 shows the throughput performance of SW-SW pairing under iSIC and HWDs for different *L* values. Table 7 shows that as HWD increases, the SNR requirement increases. These findings reveal a significant relationship: as HWD grows, the required SNR for optimal performance also increases. This trend emphasizes the importance of considering HWD conditions when optimizing system performance. From Table 7, we see that an increase in the effect of HWD leads to a degradation in throughput performance. To mitigate the losses from iSIC and HWD conditions, a large *L* is utilized, resulting in a significant enhancement in throughput performance. At *d* = 0.08, the SNR gain recorded with *L* = 256 ranges from ∼14.04 to ∼20.91 dB with *L* = 64 and 32, respectively. At *d* = 0.09, the SNR gain recorded withL = 256 ranges from ∼14.01 to ∼20.76 dB with *L* = 64 and 32, respectively. Similarly, at *d* = 0.15, the SNR gain recorded with L = 256 ranges from ∼14.03 to ∼21.02 dB with *L* = 64 and 32, respectively. This shows the significance of the largeL in the system.

**Figure 13:**
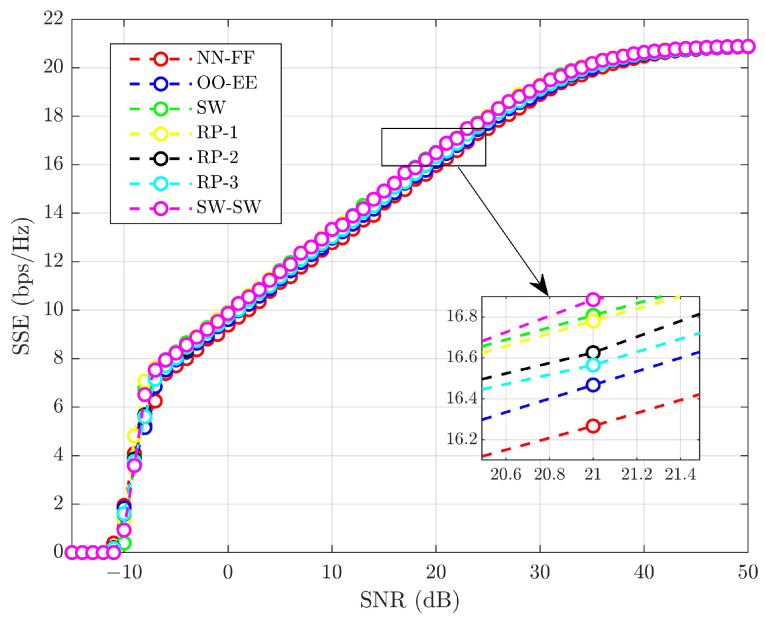
SSE comparison of varying pairing schemes.

Based on PA factors in (38) and (39), the maximum SSE of the system is determined while satisfying the QoS requirements for each UE. The SSE is plotted under various pairing schemes for *L* = 32 using the allocated power values, as shown in Figure 13. Figure 13 clearly indicates that the SW-SW pairing achieves superior SSE performance over other pairing schemes. This enhancement is due to the diverse channel gains within each pair in the sub-frames. At an SNR of 20 dB, the performance metrics are as follows: NN-FF, OO-EE, SW, RP-1, RP-2, RP-3, and SW-SW yields SSEs of ∼16.27, ∼16.47, ∼16.57, ∼16.63, ∼16.78, ∼16.81, and ∼16.89 bps/Hz, respectively. SW-SW pairing offers an SSE improvement ranging from ∼0.48% to ∼3.81% compared to other pairing schemes.

**Figure 14:**
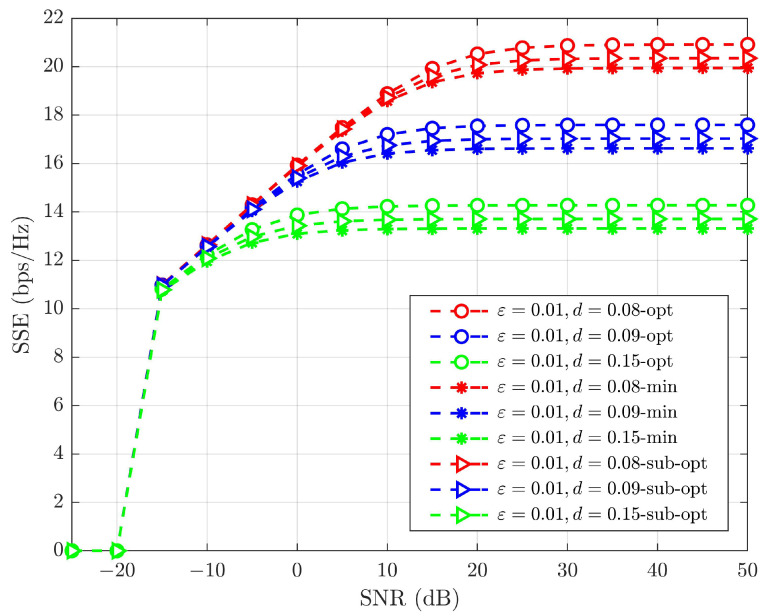
SSE of SW-SW pairing scheme with various PA strategies considering ε=0.01 and varying HWD conditions.

**Figure 15:**
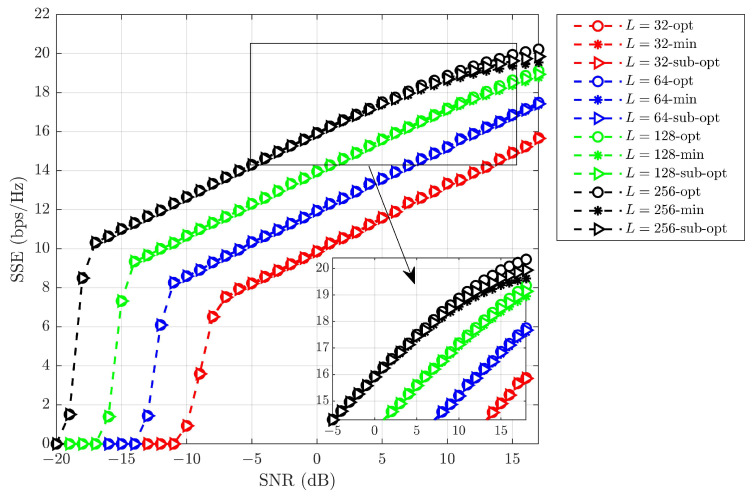
SSE (bps/Hz) comparison between optimal, sub-optimal, and minimum limit cases for varying *L* and ε=0.01 of iSIC and d=0.08 of HWDs.

Figure 14 illustrates the SSE achieved by optimizing power fractions in a SW-SW pairing scheme with L=256. To account for the SIC error and variations in HWD, the SSE values for different PA schemes were evaluated. The optimal power fractions of the SUE and WUE are described in (38) and (39), respectively. Any value between $A_{s,p}^{min}$ and $A_{s,p}^{max}$ indicates sub-optimal power allocated to the SUE. In our simulations, we assign the average of the minimum and maximum limits of (37), $A_{s,p}^{min}$ and $A_{s,p}^{max}$, to the SUE, while the remaining power is designated for the WUE. To evaluate the effectiveness of these optimal power fractions, we also use the minimum value from Equation (37) for the SUE, with the leftover power allocated to the WUE. Improvements in the SSE with these optimal power fractions are illustrated in Figure 14 and Figure 15. Simulations indicate that optimal power fractions consistently yield the highest SSE, even under conditions of HWD and SIC errors. This proves the effectiveness of optimal power fractions for maximizing the SSE, even under SIC error and HWDs.

The impact of PA strategies in SSE under an SIC error of 0.01 and varying HWD is summarized in Table 8. The optimal PA produces maximum SSE compared to other PA schemes. The power fraction is the only adjustable parameter that can be optimized to maximize SSE without triggering an outage. Both the pieces of paired UE can meet their minimum QoS requirements by optimizing the power fraction while maximizing the overall system’s SSE. Using sub-optimal PA, the SUE always allocated power less than the optimal PA power allotted. This indicates a degradation in performance. However, for applications that compromise performance for complexity, sub-optimal PA strategies may be considered. The minimum limit of PA used in the system evaluates the performance and significance of PA schemes. This minimum limit provides the least PA values, resulting in the smallest power fraction for the SUE while allocating the remaining to the WUE. Consequently, the SUE’s QoS requirement is not fulfilled. This infers that the optimal PA maximizes the SSE by fulfilling the QoS requirements of individual pieces of UE in the proposed IOS-aided C-HNOMA system.

In Figure 15, the SSE for optimal, sub-optimal, and minimum limits is compared by varying the parameter *L* and introducing a 1% SIC error, along with an HWD of d= 0.08. As *L* increases, the SSE improves across all PA schemes. Table 9 shows the impact of optimal power values on SSE for different values of *L* at an SNR of 15 dB. The table clearly shows that the influence of optimal PA results in maximum SSE for larger values of *L*. For smaller values of *L*, the effect of optimal PA compared to other PA schemes is less pronounced. However, as *L* increases, optimal PA leads to a significant improvement in SSE over other PA schemes. This indicates that the optimal power fraction is ideal for achieving maximum SSE, while the sub-optimal fraction is more suitable for less complex systems. The choice between optimal and sub-optimal values reflects a trade-off between system complexity and performance.

**Figure 16:**
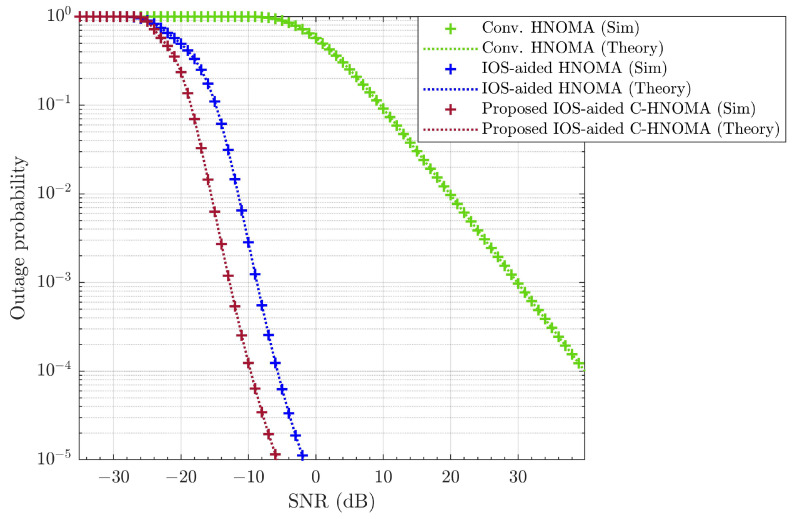
Average outage probability comparison among the proposed IOS-aided C-HNOMA, conventional HNOMA, and IOS-aided HNOMA schemes with SW-SW pairing for ε=1% and d=0.08.

**Figure 17:**
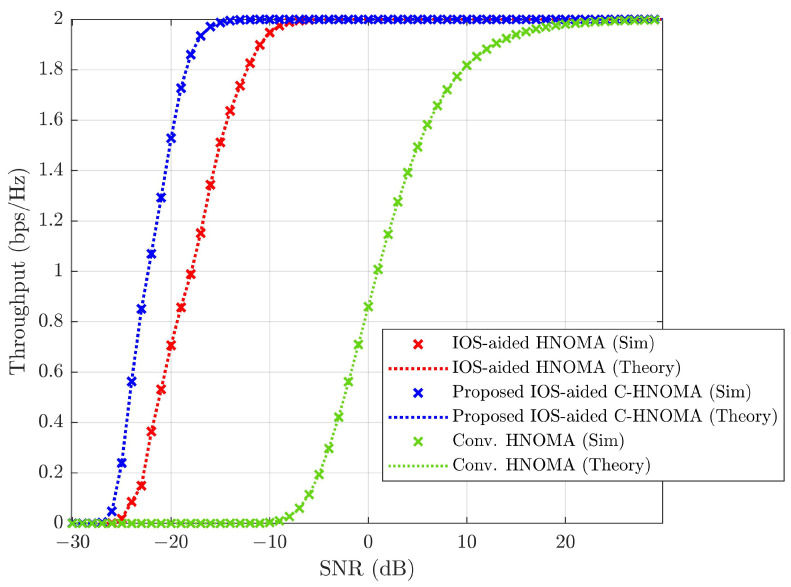
Throughput (bps/Hz) comparison among the proposed IOS-aided C-HNOMA, conventional HNOMA, and IOS-aided HNOMA schemes with SW-SW pairing for ε=1% and d=0.08.

**Figure 18:**
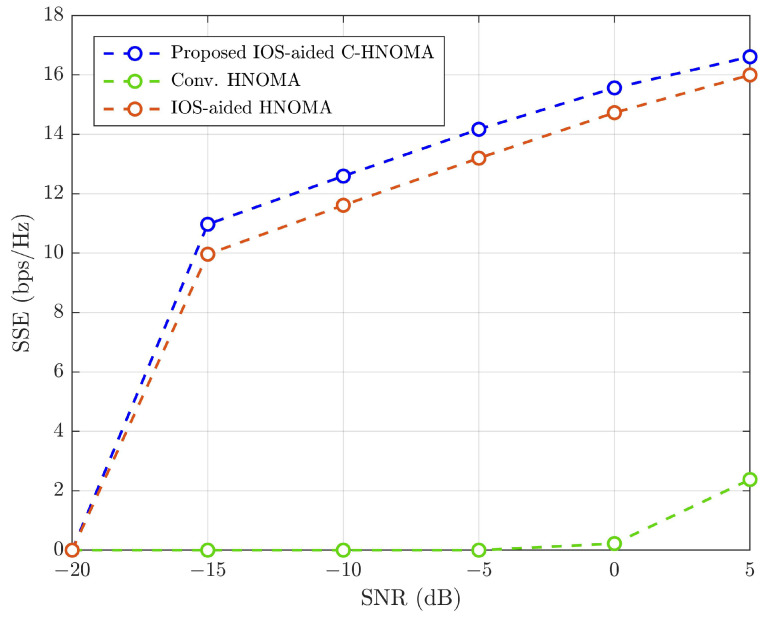
SSE (bps/Hz) comparison among the proposed IOS-aided C-HNOMA, conventional HNOMA, and IOS-aided HNOMA schemes with SW-SW pairing for ε=1% and d=0.08.

Simulations assess the effects of varying conditions on wireless link performance, focusing on outage probability, throughput, and SSE. The analysis compares the proposed IOS-aided C-HNOMA system with conventional HNOMA [6] and IOS-aided HNOMA [16] to evaluate performance improvements. The simulation results show that the SW-SW pairing scheme outperforms other pairing schemes, and the optimal power fraction achieves maximum SSE. By fixing SW-SW pairing and optimal power allocation, the proposed IOS-aided C-HNOMA system is compared with conventional HNOMA and IOS-aided HNOMA systems. Figure 16 illustrates the average outage probability, Figure 17 presents the throughput performance, and Figure 18 shows the SSE of eight UEs under the proposed and conventional systems. All performance metrics—outage probability, throughput, and SSE—are evaluated under an iSIC condition of 1% and an HWD of d=0.08. The results demonstrate that integrating IOSs into hybrid NOMA significantly improves system performance, while retransmission for the WUE or cell-edge UE in cooperative communication further enhances overall system efficiency. The integration of IOSs enhances system reliability, and cooperative communication improves the performance of the WUE in each pair, leading to better overall performance. According to Figure 16, achieving a target outage of $10^{-3}$ requires ∼30 dB of SNR for conventional HNOMA. In contrast, IOS-aided HNOMA requires ∼−9dB of SNR, while the proposed IOS-aided C-HNOMA requires only ∼−13 dB of SNR. This results in an SNR gain of ∼4 to ∼43 dB with the proposed solution. In Figure 17, to attain a throughput of 2 bps/Hz, conventional HNOMA demands ∼20 dB of SNR, whereas IOS-aided HNOMA needs ∼−8 dB of SNR. The proposed IOS-aided C-HNOMA, on the other hand, requires only ∼−14 dB of SNR, offering an SNR gain of ∼6 to ∼34 dB. Lastly, Figure 18 shows that, at an SNR of 5 dB, conventional HNOMA achieves ∼2.38 bps/Hz, while IOS-aided HNOMA reaches ∼15.98 bps/Hz of SSE. The proposed IOS-aided C-HNOMA achieves ∼16.71 bps/Hz of SSE. This results in a difference of ∼0.73 to ∼14.33 bps/Hz in SSE with the proposed solution. Thus, the proposed IOS-aided C-HNOMA outperforms both conventional HNOMA and IOS-aided HNOMA, demonstrating its significant advantages over existing NOMA variants. By effectively addressing the limitations of traditional HNOMA and IOS-aided HNOMA, the IOS-aided C-HNOMA system proves to be a strong candidate for future wireless networks requiring high SSE, throughput, and reliable connectivity.

## 6. Conclusions

In conclusion, this paper emphasizes the potential benefits of integrating IOSs into C-HNOMA systems to address challenges associated with iSIC and HWDs. The system model is developed for multi-user systems, and the analytical expressions for average outage probability, throughput, and SSE are derived and validated through simulations. The optimization framework for PA has been developed to maximize the SSE. The proposed model demonstrates significant improvements in outage probability, throughput, and SSE performance. The pairing method in the proposed systems enhances overall performance. Specifically, the SW-SW pairing offers an SNR gain ranging from ∼0.3 to ∼3.81 dB for the SUE and ∼0.21 to ∼3.89 dB for the WUE against other pairing schemes. Additionally, SW-SW pairing consistently shows improved throughput performance, with an increase in SSE of ∼0.48% to ∼3.81% compared to other pairing schemes. By optimizing the PA values, with a fixed SIC error of 0.01 and an HWD of 8%, SSE sees ∼2.24% to ∼4.06% improvement compared to other PA factors. As the number of IOS components increases, significant improvements are observed even in the presence of iSIC and HWDs.

These promising results highlight the importance of incorporating IOSs for enhanced performance and scalability in future wireless networks. Future research could focus on optimizing IOS configurations using machine learning, integrating IOSs with advanced technologies such as mmWave communications and massive MIMO, and examining strategies for user pairing and PA to further improve system robustness and efficiency.

## Figures and Tables

**Table 1:** Comparison of proposed work with existing studies.

Ref.	Multiple UE	HNOMA	C-NOMA	IOS/RIS	Outage Probability	Outage Probability Under iSIC and HWDs	Optimization	Throughput	Throughput Under iSIC and HWDs	SSE	SSE Under iSIC and HWDs
[3]	✓	×	×	×	×	×	✓	✓	×	✓	×
[22]	×	×	×	×	✓	×	✓	×	×	✓	×
[6]	✓	✓	×	×	✓	×	✓	×	×	✓	×
[23]	✓	×	×	×	×	×	✓	✓	×	✓	×
[7]	×	×	✓	×	×	×	✓	×	×	✓	✓
[8]	×	×	✓	×	✓	×	✓	✓	×	✓	×
[9]	✓	×	✓	×	×	×	×	✓	×	✓	×
[24]	×	×	✓	×	×	×	×	×	✓	✓	×
[4]	×	×	×	×	✓	✓	✓	✓	✓	✓	✓
[5]	✓	✓	×	×	✓	✓	×	×	×	✓	×
[10]	×	×	×	RIS	✓	×	×	×	×	×	×
[15]	✓	×	×	IOS	×	×	×	×	×	✓	×
[11]	✓	✓	×	RIS	×	×	✓	×	×	×	×
[25]	✓	✓	×	RIS	×	×	×	✓	×	✓	×
[26]	×	✓	×	RIS	✓	✓	×	✓	✓	×	×
[12]	✓	×	×	RIS	✓	×	×	×	×	✓	×
[13]	✓	✓	×	RIS	✓	×	×	×	×	✓	×
[14]	×	×	×	IOS	×	×	×	✓	×	✓	×
[16]	✓	✓	×	IOS	×	×	×	×	×	✓	×
[20]	✓	×	×	IOS	×	×	×	×	×	✓	×
This work	✓	✓	✓	IOS	✓	✓	✓	✓	✓	✓	✓

**Table 2:** UEs paired in every sub-frame for various pairing strategies.

Sub-Frame	NN-FF	OO-EE	SW-SW	SW	RP-1	RP-2	RP-3
i=1	$UE_{1}$ and $UE_{2}$	$UE_{1}$ and $UE_{3}$	$UE_{1}$ and $UE_{5}$	$UE_{1}$ and $UE_{8}$	$UE_{1}$ and $UE_{4}$	$UE_{1}$ and $UE_{6}$	UE_1_ and UE_7_
i=2	$UE_{3}$ and $UE_{4}$	$UE_{2}$ and $UE_{4}$	$UE_{2}$ and $UE_{6}$	$UE_{2}$ and $UE_{7}$	$UE_{2}$ and $UE_{5}$	$UE_{2}$ and $UE_{3}$	$UE_{2}$ and $UE_{8}$
i=3	$UE_{5}$ and $UE_{6}$	$UE_{5}$ and $UE_{7}$	$UE_{3}$ and $UE_{7}$	$UE_{3}$ and $UE_{6}$	$UE_{3}$ and $UE_{8}$	$UE_{4}$ and $UE_{7}$	$UE_{3}$ and $UE_{5}$
i=4	$UE_{7}$ and $UE_{8}$	$UE_{6}$ and $UE_{8}$	$UE_{4}$ and $UE_{8}$	$UE_{4}$ and $UE_{5}$	$UE_{6}$ and $UE_{7}$	$UE_{5}$ and $UE_{8}$	$UE_{4}$ and $UE_{6}$

**Table 3:** Definition of symbols and notations.

Notation	Description
$x_{u}$	Symbol corresponding to *u*th UE
*p*	Index of pairing scheme (NN-FF, OO-EE, SW-SW, SW, and RP)
$X_{p}^{i}$	Composite signal transmitted at time slot,i for pairing scheme, *p*
*P*	Power budget
$\mu_{si},\mu_{wi}$	Power fractions of SUE and WUE
*M*	Total number of UE
*T*	Total frame duration
$T_{i}$, i=1,2,…,M/2	*i*th sub-frame duration
$\delta_{u}^{2}$	Channel gain of *u*th UE
$n_{u}$	Additive white Gaussian noise (AWGN) added touth UE with noise power $\sigma_{u}^{2}$
$k_{g}$	HWDs from gNB
$k_{s}$	HWDs from SUE
$k_{w}$	HWDs from WUE
$w_{s}$	Hardware noise at SUE during direct transmission, $w_{s}\;\epsilon \;CN\left(0,\left(k_{g}^{2}+k_{s}^{2}\right)\right)$ [29,30]
$w_{w}$	Hardware noise at WUE during direct transmission, $w_{w}\epsilon \;CN\left(0,\left(k_{g}^{2}+k_{w}^{2}\right)\right)$ [29,30]
$w_{c}$	Hardware noise at WUE during cooperative relaying, $w_{s}\epsilon \left(0,\left(k_{s}^{2}+k_{w}^{2}\right)\right)$ [29,30]
$y_{d-u}^{r,f}$	Received signal at UE either from reflecting side or refractive side of IOS through direct transmission
$y_{c-u}^{r,f}$	Received signal at UE either from reflecting side or refractive side of IOS through cooperative relaying
$h_{d}$	Channel between gNB and IOS during direct transmission
$g_{d-u}$	Channel between IOS and UE during direct transmission
$h_{c-u}$	Channel between SUE and IOS during cooperative relaying
$g_{c-u}$	Channel between IOS and WUE during cooperative relaying
$\Theta^{r,f}$	IOS phase shift matrix
$\theta_{l\;}\epsilon \left(0,2\pi \right]$	Phase compensation produced by *l*th IOS component
$\alpha_{l}$ and $\psi_{l}$	Magnitude of channel coefficient and phase angle between gNB and *l*th IOS component
$\beta_{l}$ and $\varphi_{l}$	Magnitude of channel coefficient and phase angle between *l*th IOS component and UE
$\chi_{l}^{r,f}\epsilon \left(0,1\right)$	Coefficient of *l*th IOS component for reflection/refraction side
$H_{u}$	Cumulative channel observed at UE located in reflecting/refractive side of IOS
ρ	Instantaneous SNR
ε	SIC error
$C_{p}^{i}$	SSE at *i*th time slot of *p*th pairing scheme
$\Gamma_{d-s,p}^{i}$	SE of the SUE at *i*th time slot of *p*th pairing scheme during direct transmission
$\Gamma_{d-w,p}^{i}$	SE of the WUE at *i*th time slot of *p*th pairing scheme during direct transmission
$\Gamma_{d-ws,p}^{i}$	SE of WUE detected at the SUE at *i*th time slot of *p*th pairing scheme during direct transmission
$\Gamma_{c-w,p}^{i}$	SE of the WUE at *i*th time slot of *p*th pairing scheme during cooperative relaying
${\tilde{R}}_{u}$	Minimum SE requirement *u*th UE
$P_{s}$	Average outage probability of SUE
$P_{w}$	Average outage probability of WUE
*L*	Total number of IOS components
$L_{u}$	Number of IOS components dedicated to *u*th UE
P(.)	Probability function
Γ(.)	Gamma function
$\gamma \left(.\right)$	Incomplete Gamma function

**Table 4:** Simulation set-up.

Simulation Parameters	Values
Block length	$10^{7}$
Number of UE, *M*	8
Power fractions during direct relaying, $\mu_{si}$ and $\mu_{wi}$ [4]	0.1 and 0.9
Path-loss exponent, η (sub-urban region)	3.8
Channel gains of UE, $\delta_{u}^{i}$, u=1,2,…,8 [12]	8, 7, 6, 5, 4, 3, 2 and 1
Target SE of SUE, ${\tilde{R}}_{s,p}^{i}$ [31]	0.5 bps/Hz, 1 bps/Hz
Target SE of WUE, ${\tilde{R}}_{w,p}^{i}$ [31]	0.5 bps/Hz, 1 bps/Hz
Threshold SNR of SUE, $\rho_{s,p}^{i}$ [29]	15 dB
Threshold SNR of WUE, $\rho_{w,p}^{i}$ [29]	15 dB
Target ouatge	$10^{-3}$ and $10^{-5}$
Noise variance, $\sigma_{u}^{2}$	1
SIC error, ε [4]	1%
HWD, $d=k_{g}^{2}=k_{s}^{2}=k_{w}^{2}=k_{u}$ [30]	0.08, 0.09 and 0.15
Number of IOS elements, *L* [12,32]	16, 32, 64 128 and 256

**Table 5:** SNR (dB) gain with SW-SW pairing compared to other pairing schemes to achieve the target outage probability of $10^{-3}$.

Pairing	SUE		WUE	
	SNR (dB) Required	SNR Gain (dB) w.r.t SW-SW Scheme	SNR (dB) Required	SNR Gain (dB) w.r.t SW-SW Scheme
NN-FF	∼−8.10	∼3.81	∼−15.41	∼3.89
OO-EE	∼−9.56	∼2.35	∼−16.62	∼2.68
SW	∼−9.80	∼2.11	∼−17.57	∼1.73
RP-1	∼−10.68	∼1.23	∼−17.65	∼1.65
RP-2	∼−11.43	∼0.48	∼−18.33	∼0.97
RP-3	∼−11.61	∼0.3	∼−19.09	∼0.21
SW-SW	∼−11.91	-	∼−19.30	-

**Table 6:** SNR requirement for the SUE and WUE to reach outage of $10^{-5}\;$under various *L* and HWDs.

*L*	SUE (ε=0.01)			WUE		
	d=0.08	d=0.09	d=0.15	d=0.08	d=0.09	d=0.15
32	∼−8.05	∼−4.22	∼−1.18	∼−22.65	∼−19.54	∼−16.36
64	∼−22.03	∼−18.17	∼−15.12	∼−31.77	∼−28.68	∼−25.49
256	∼−38.67	∼−34.71	∼−31.67	∼−46.52	∼−43.43	∼−40.23

**Table 7:** SNR requirement for SW-SW pairing to reach throughput of 1 bps/Hz with ε=0.01 under various *L* and HWDs.

*L*	SNR (dB) Required to Reach a Throughput of 1 bps/Hz		
	d=0.08	d=0.09	d=0.15
32	∼−22	∼−19.76	∼−17.20
64	∼−28.87	∼−26.72	∼−24.19
256	∼−42.91	∼−40.73	∼−38.22

**Table 8:** Comparison of the SSE with ε=0.01 under HWDs with L=256.

*d*	SSE (bps/Hz) at 20 dB of SNR			% Improvement with Optimal PA w.r.t Sub-Optimal PA	% Improvement with Optimal PA w.r.t Minimum Limit PA
	Optimal	Sub-Optimal	Minimum Limit		
0.08	∼20.53	∼20.08	∼19.73	∼2.24%	∼4.06%
0.09	∼17.55	∼16.99	∼16.60	∼3.29%	∼5.72%
0.15	∼14.27	∼13.71	∼13.31	∼4.09%	∼7.21%

**Table 9:** Comparison of SSE (bps/Hz) for optimal, sub-optimal, and minimum limit cases for varying Lat 15 dB SNR.

PA Scheme/*L*	32	64	128	256
Optimal	∼14.92	∼16.86	∼18.63	∼19.94
Sub-optimal	∼14.90	∼16.82	∼18.49	∼19.62
Minimum limit	∼14.88	∼16.78	∼18.37	∼19.37
Difference between optimal and sub-optimal (bps/Hz)	∼0.02	∼0.04	∼0.14	∼0.32
Difference between optimal and minimum limit (bps/Hz)	∼0.04	∼0.08	∼0.26	0.57

## Data Availability

Data sharing not applicable to this article as no datasets were generated or analyzed during the current study.

## Abbreviations

The following abbreviations are used in this manuscript:

AO	Alternate optimization
BER	Bit error rate
C-HNOMA	Cooperative hybrid NOMA
C-NOMA	Cooperative NOMA
5G	Fifth-generation
HWD	Hardware distortion
HNOMA	Hybrid NOMA
iSIC	Imperfect successive interference cancellation
ISAC	Integrated sensing and communication
IOS	Intelligent omni-surfaces
IoT	Internet of things
LoS	Line-of-sight
mmWave	Millimeter wave
MIMO	Multi-input multi-output
NN-FF	Near–near and far–far
NOMA	Non-orthogonal multiple access
OO-EE	Odd–odd and even–even
OMA	Orthogonal multiple access
PA	Power allocation
RP	Random pairing
SNR	Signal-to-noise ratio
6G	Sixth-generation
SW-SW	Stronger–weak and strong–weak
SW	Strong–weak
SIC	Successive interference cancellation
SSE	Sum spectral efficiency
UE	User equipment
V2V	Vehicle-to-vehicle
V2X	Vehicle-to-everything

## Appendix A

The expression for the probability of outage at the SUE during direct transmission is given by

(A1) $$P_{s,p}^{i}=P\left(\Gamma_{d-ws,p}^{i}<{\tilde{R}}_{w,p}^{i}\;,\Gamma_{d-s,p}^{i}<{\tilde{R}}_{s,p}^{i}\right)$$

Substituting (7) and (3) in (A1) gives

(A2) $$P_{s,p}^{i}=P\left(\frac{1}{M}\log_{2}\left(1+\frac{G_{s}\rho \mu_{wi}}{G_{s}\rho \mu_{si}+G_{s}\rho \left(k_{g}^{2}+k_{s}^{2}\right)+1}\right)<{\tilde{R}}_{w,p}^{i}\;,\frac{1}{M}\log_{2}\left(1+\frac{G_{s}\rho \mu_{si}}{\varepsilon G_{s}\rho \mu_{wi}+G_{s}\rho \left(k_{g}^{2}+k_{s}^{2}\right)+1}\right)<{\tilde{R}}_{s,p}^{i}\right)$$

Taking antilog within the conditions in (A2) gives

(A3) $$P_{s,p}^{i}=P\left(\left(\frac{G_{s}\rho \mu_{wi}}{G_{s}\rho \mu_{si}+G_{s}\rho \left(k_{g}^{2}+k_{s}^{2}\right)+1}\right)<\rho_{w,p}^{i}\right)\;\times P\left(\left(\frac{G_{s}\rho \mu_{si}}{\varepsilon G_{s}\rho \mu_{wi}+G_{s}\rho \left(k_{g}^{2}+k_{s}^{2}\right)+1}\right)<\rho_{s,p}^{i}\right)$$

Solving (A3) for the channel condition $H_{s,p}^{i}$ gives Equation (11).

## Appendix B

The expression for the probability of outage at the WUE during direct transmission is given by

(A4) $$P_{w,p}^{i}=P\left(\Gamma_{d-w,p}^{i}<{\tilde{R}}_{w,p}^{i}\right)$$

Substituting (5) in (A4) gives

(A5) $$P_{w,p}^{i}=P\left(\frac{1}{M}\log_{2}\left(1+\frac{G_{w}\rho \mu_{wi}}{G_{w}\rho \mu_{si}+G_{w}\rho \left(k_{g}^{2}+k_{w}^{2}\right)+1}\right)<{\tilde{R}}_{w,p}^{i}\right)$$

Taking antilog within the condition in (A5) gives

(A6) $$P_{w,p}^{i}=P\left(\left(\frac{G_{w}\rho \mu_{wi}}{G_{w}\rho \mu_{si}+G_{w}\rho \left(k_{g}^{2}+k_{w}^{2}\right)+1}\right)<\rho_{w,p}^{i}\right)$$

Solving (A6) for the channel condition $H_{w,p}^{i}$ gives Equation (16).

## Appendix C

The expression for the probability of outage at the WUE during cooperative relaying is given by,

(A7) $$P_{c-w,p}^{i}=P\left(\Gamma_{c-w,p}^{i}<{\tilde{R}}_{w,p}^{i}\right)$$

Substituting (9) in (A7) gives,

(A8) $$P_{c-w,p}^{i}=P\left(\frac{1}{M}\log_{2}\left(1+\frac{G_{c-p}\rho }{G_{c-p}\rho \left(k_{s}^{2}+k_{w}^{2}\right)+1}\right)<{\tilde{R}}_{w,p}^{i}\right)$$

Taking antilog within the condition in (A8) gives

(A9) $$P_{c-w,p}^{i}=P\left(\left(\frac{G_{c-p}\rho }{G_{c-p}\rho \left(k_{s}^{2}+k_{w}^{2}\right)+1}\right)<\rho_{w,p}^{i}\right)$$

Solving (A9) for the channel condition $H_{c-w,p}^{i}$ gives the expression (21).
